# AA16 Oxidoreductases Boost Cellulose-Active AA9 Lytic Polysaccharide Monooxygenases from *Myceliophthora thermophila*

**DOI:** 10.1021/acscatal.3c00874

**Published:** 2023-03-21

**Authors:** Peicheng Sun, Zhiyu Huang, Sanchari Banerjee, Marco A. S. Kadowaki, Romy J. Veersma, Silvia Magri, Roelant Hilgers, Sebastian J. Muderspach, Christophe V.F.P. Laurent, Roland Ludwig, David Cannella, Leila Lo Leggio, Willem J. H. van Berkel, Mirjam A. Kabel

**Affiliations:** † Laboratory of Food Chemistry, Wageningen University & Research, Bornse Weilanden 9, 6708 WG Wageningen, The Netherlands; ‡ Department of Chemistry, University of Copenhagen, Universitetsparken 5, 2100 Copenhagen, Denmark; § PhotoBioCatalysis Unit (CPBL) and Biomass Transformation Lab (BTL), École Interfacultaire de Bioingénieurs (EIB), Université Libre de Bruxelles, Avenue Franklin D. Roosevelt 50, 1050 Bruxelles, Belgium; ∥ Biocatalysis and Biosensing Laboratory, Department of Food Science and Technology, University of Natural Resources and Life Sciences (BOKU), Muthgasse 18, 1190 Vienna, Austria; ⊥ Institute of Molecular Modeling and Simulation, Department of Material Sciences and Process Engineering, University of Natural Resources and Life Sciences (BOKU), Muthgasse 18, 1190 Vienna, Austria

**Keywords:** cellulose, Carbohydrate-Active enZyme, copper-dependent oxidoreductase, fungal auxiliary activity family, hydrogen peroxide, lytic polysaccharide monooxygenase, protein structure

## Abstract

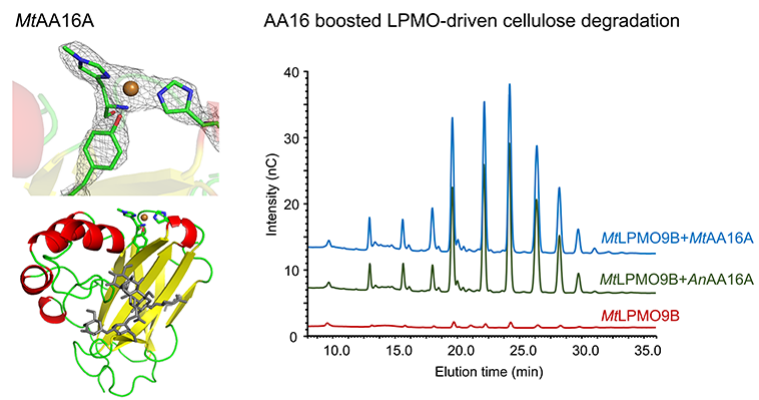

Copper-dependent lytic polysaccharide monooxygenases (LPMOs) classified in Auxiliary Activity (AA) families are considered indispensable as synergistic partners for cellulolytic enzymes to saccharify recalcitrant lignocellulosic plant biomass. In this study, we characterized two fungal oxidoreductases from the new AA16 family. We found that *Mt*AA16A from *Myceliophthora thermophila* and *An*AA16A from *Aspergillus nidulans* did not catalyze the oxidative cleavage of oligo- and polysaccharides. Indeed, the *Mt*AA16A crystal structure showed a fairly LPMO-typical histidine brace active site, but the cellulose-acting LPMO-typical flat aromatic surface parallel to the histidine brace region was lacking. Further, we showed that both AA16 proteins are able to oxidize low-molecular-weight reductants to produce H_2_O_2_. The oxidase activity of the AA16s substantially boosted cellulose degradation by four AA9 LPMOs from *M. thermophila* (*Mt*LPMO9s) but not by three AA9 LPMOs from *Neurospora crassa* (*Nc*LPMO9s). The interplay with *Mt*LPMO9s is explained by the H_2_O_2_-producing capability of the AA16s, which, in the presence of cellulose, allows the *Mt*LPMO9s to optimally drive their peroxygenase activity. Replacement of *Mt*AA16A by glucose oxidase (*An*GOX) with the same H_2_O_2_-producing activity could only achieve less than 50% of the boosting effect achieved by *Mt*AA16A, and earlier *Mt*LPMO9B inactivation (6 h) was observed. To explain these results, we hypothesized that the delivery of AA16-produced H_2_O_2_ to the *Mt*LPMO9s is facilitated by protein–protein interaction. Our findings provide new insights into the functions of copper-dependent enzymes and contribute to a further understanding of the interplay of oxidative enzymes within fungal systems to degrade lignocellulose.

## 1 Introduction

Transition from a fossil-based society to a more sustainable one drives full valorization of lignocellulose-rich agricultural and forestry side-streams for the production of biofuels, biomaterials, and biochemicals.^1^ Hereto, enzyme-driven degradation of cellulose and hemicellulose to fermentable monosaccharides is an essential step,^2^ in which copper-dependent lytic polysaccharide monooxygenases (LPMOs) are key. LPMOs have been shown to oxidatively cleave in particular insoluble substrates, such as cellulose, which synergistically enhances cellulose saccharification by established cellulases. As such LPMOs have become a permanent ingredient in cellulolytic enzyme formulations.^3−5^ LPMOs are currently classified as “Auxiliary Activity” (AA) families 9–11 and 13–17 in the Carbohydrate-Active enZymes (CAZy) database (http://www.cazy.org).^6,7^

**Figure 1:**
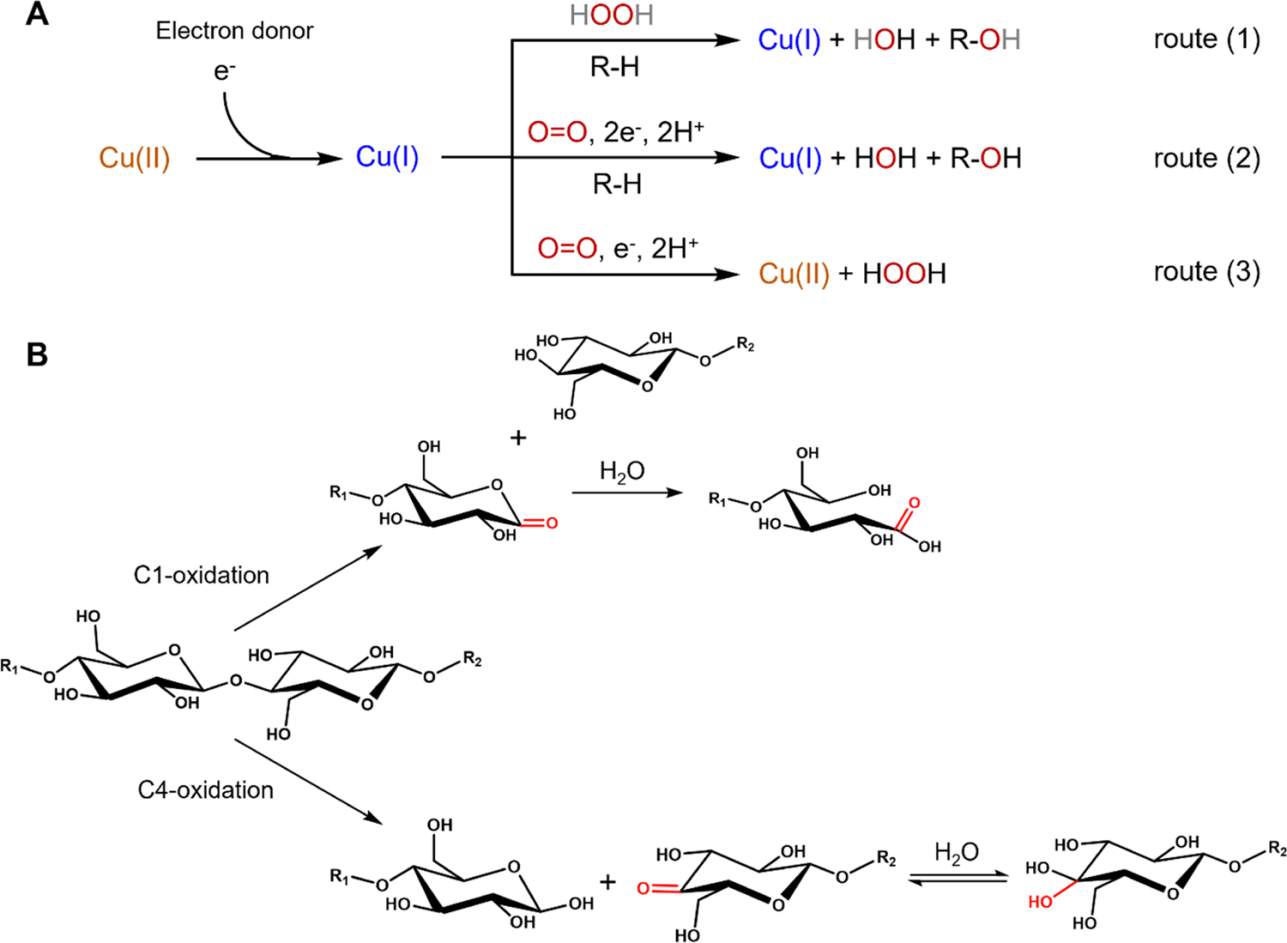
(A) Proposed catalytic routes for LPMO reactions using H_2_O_2_ or O_2_. In both H_2_O_2_- and O_2_-dependent routes, the C1- and/or C4-carbon position of the carbohydrate (R–H) substrate is hydroxylated (R–OH) and a Cu(I) is ready for the next catalytic cycle. The oxidation at the C1-carbon leads to the formation of δ-lactone, which is converted to an aldonic acid in water. The oxidation at C4-carbon results the generation of 4-ketoaldose, which is in pH-dependent equilibrium with the geminal diol. Adapted from Wang et al.^21^ and Chylenski et al.^25^

To explore the AA diversity in nature and improve enzyme formulations, new AA families with enigmatic functions need further investigation.^8−10^ The recently proposed AA16 family contains so far only one characterized member from *Aspergillus aculeatus* (*Aa*AA16).^11^*Aa*AA16 has been indicated as C1-cellulose-active LPMO,^11^ though its activity is much lower compared to accustomed C1-oxidizing AA9 LPMOs (Figure 1).^12,13^

Based on sequence comparison, AA16 members were reported to share common features with other LPMOs, for instance, a copper-dependent active site coordinated by two histidines and a tyrosine (sometimes a phenylalanine residue in AA10 LPMOs).^14^ This coordination, also referred to as “histidine brace” (His-brace), is conserved in all LPMOs.^14,15^ Although the catalytic mechanism of LPMOs is not fully clear, it is well accepted that the catalytic cycle starts with a so-called “priming reduction” of Cu(II) to Cu(I) by an external electron donor.^16^ The external electron donor can be a chemical reductant, such as ascorbic acid (Asc), phenolics (e.g., pyrogallol (Pyg)) including lignin, or a redox enzyme as is well established for cellobiose dehydrogenase (CDH).^17,18^ After this priming reduction, the catalytic reaction can follow two routes depending on the cosubstrate, which can either be H_2_O_2_ or O_2_ (Figure 1, route 1 or 2).^16,19,20^ The mechanistic details of these routes are still under debate as extensively reviewed elsewhere.^21,22^ In the absence of a carbohydrate substrate, superoxide (or hydroperoxide) is released, resulting in the production of H_2_O_2_ and regeneration of the Cu(II) state (Figure 1, route 3).^13^

H_2_O_2_ has been shown to be the preferred cosubstrate over O_2_, as turnover numbers obtained with H_2_O_2_ are in certain cases more than three orders of magnitude higher compared to those obtained with O_2_.^16,20,23^ On the other hand, a high H_2_O_2_ concentration induces oxidative damage of amino acids close to the copper-active site resulting in self-inactivation. Hence, the optimal H_2_O_2_ concentration is a balance between activity and inactivation and upholds a delicate equilibrium in LPMO reactions.^16,20,23,24^

H_2_O_2_ typically results from nonenzymatic or enzymatic routes to drive LPMO reactions. Nonenzymatic H_2_O_2_ formation results from metal ions reduced by molecular reductants (e.g., Asc, cysteine) and subsequently reacting with dissolved O_2_,^26,27^ while some H_2_O_2_-producing oxidases (e.g., glucose oxidase) also can take up that role.^16^ It is noteworthy that, as mentioned above, in absence of a carbohydrate substrate, (reduced) LPMOs show oxidase activity to produce H_2_O_2_. In a recent study, Stepnov and co-workers described that a weak cellulose-binding *Sc*LPMO10C_TR_ (only catalytic domain, without a linker and a carbohydrate binding module) served as a H_2_O_2_ producer to enhance oxidative cellulose cleavage by full-length *Sc*LPMO10C.^28^

Here, we characterized two new members of the AA16 family. *Mt*AA16A was homologously produced in *Myceliophthora thermophila* C1, while *An*AA16A from *Aspergillus nidulans* was produced in *Pichia pastoris* X-33. In contrast to the published *Aa*AA16, both *Mt*AA16A and *An*AA16A did not oxidatively cleave carbohydrate substrates. We elucidated the crystal structure of *Mt*AA16A and showed that both AA16s display oxidase activity. Furthermore, we found a substantial boosting effect of the AA16s on various well-characterized *Mt*LPMO9s in degrading cellulose. This boosting effect was absent when the AA16s were combined with three distinct and well-studied AA9 *Neurospora crassa* (*Nc*) LPMOs. We discuss possible reasons for this observed (lack of) interplay. In summary, we suggest that AA16 proteins are H_2_O_2_-producing oxidoreductases that may assist LPMOs in degrading lignocellulose.

## 2 Experimental Section

### 2.1 Materials

Syringol, pyrogallol, and ammonium acetate were purchased from Sigma-Aldrich (St. Louis, Missouri). Cellobiose, cellotriose, cellotetraose, cellopentaose, and cellohexaose were purchased from Megazyme (Bray, Ireland). Regenerated amorphous cellulose (RAC) was prepared from Avicel PH-101 (Sigma-Aldrich) as described previously.^29^ Ascorbic acid (Asc) was purchased from VWR International (Radnor, Pennsylvania). Other aromatic compounds used were purchased from Sigma-Aldrich or VWR International. Other carbohydrate substrates used were purchased from Sigma-Aldrich or Megazyme. Glucose oxidase from *Aspergillus niger* (*An*GOX, 10 000 U g^–1^ powder) was purchased from Sigma-Aldrich. All water used was produced by a Milli-Q system (Merck Millipore, Molsheim, France), unless stated otherwise.

### 2.2 Expression, Production, and Purification of *Mt*AA16A and *An*AA16A

#### 2.2.1 *Mt*AA16A

The gene encoding *Mt*AA16A (MYCTH_2306267, UniProt ID: G2QH80) was homologously expressed and produced in a low protease/low (hemi)cellulase-producing *M. thermophila* C1 strain as described elsewhere.^30,31^

*Mt*AA16A was purified by three subsequent chromatographic steps. Crude *Mt*AA16A-rich fermentation broth was filtered and dialyzed against 10 mM potassium phosphate buffer pH 7.6 before chromatographic purification. The dialyzed *Mt*AA16A was purified by anion-exchange chromatography (AEC), followed by size-exclusion chromatography (SEC). Purification settings and elution programs of AEC and SEC have been described previously.^32^ The SEC-purified *Mt*AA16A-containing fractions were further purified by cation-exchange chromatography (CEC) on an ÄKTA-Micro preparative chromatography system (GE Healthcare). *Mt*AA16A-containing fractions were loaded on a Resource S column (30 × 16 mm internal diameter, GE Healthcare) pre-equilibrated with 10 mM sodium acetate buffer pH 3.8 (eluent A). The unbound fraction was first removed (one column volume). Eluent B was 10 mM sodium acetate buffer (pH 3.8) containing 500 mM NaCl. Elution (flow rate of 1 mL min^–1^) was performed as follows: from 0 to 30% B in two column volumes; 30% B for one column volume; next, 30–100% B over two column volumes; and finally, 100% B for four column volumes. All fractions were collected and immediately stored on ice. Peak fractions (based on UV absorption at 280 nm) were adjusted to an approximate concentration of 2 mg mL^–1^ (as determined by the bicinchoninic acid method) and analyzed by sodium dodecyl sulfate-poly(acrylamide) gel electrophoresis (SDS-PAGE), as described previously,^32^ to determine the *Mt*AA16A fractions. CEC-purified *Mt*AA16A-containing fractions were combined and used as the final enzyme stock solution. All CEC-purified *Mt*AA16A fractions were aliquoted into 500 μL size and stored at −80 °C.

#### 2.2.2 *An*AA16A

The *An*AA16A gene (AN0778.2) was amplified directly from the *A. nidulans* genome and produced in a *P. pastoris* X-33 strain, as described hereafter. The oligonucleotides *An*AA16fw (5′ACAACTAATTATTCGAAACGATGAAGCACGCTACCACCG3′) and *An*AA16rv (5′CCCTGAAAATAAAGATTCTCGCCGTTACCACTTCCACCAA3′) were used to remove the *C*-terminal extension region (residues 199–306) and maintain the native signal peptide. The removal of *C*-terminal extension region prior to protein production has been reported by Filiatrault-Chastel et al.^11^ The *An*AA16 gene was cloned into a modified pPICZα vector, as previously described.^33^ This construction allowed the expression of a recombinant *An*AA16A containing a cleavable *C*-terminal polyhistidine-tag. The *P. pastoris* X-33 (Invitrogen, Waltham, Massachusetts) was transformed by electroporation using a *Pme*I-linearized plasmid (pPICZT::*An*AA16A) and selected on yeast extract-peptone-dextrose-sorbitol (YPDS)-zeocin plates. The recombinant colonies were randomly picked and grown in a buffered methanol-complex (BMMY) medium, and the gene expression was confirmed by SDS-PAGE analysis of the supernatant content. The transformant showing the highest expression profile was grown in 40 mL of YPD medium overnight and inoculated in four Erlenmeyer flasks containing 0.5 L buffered glycerol-complex medium (BMGY) medium at 30 °C and 250 rpm until an OD600 of 2. The yeast cells from each flask were harvested and transferred to 0.1 L BMMY medium and incubated at 30 °C and 250 rpm for 72 h. Two percent absolute methanol was added every 24 h to maintain recombinant protein production. The culture supernatant was filtered, and the pH was adjusted to 8.0 using the Tris–HCl buffer. The entire volume was loaded onto a 5 mL HiTrap Chelating HP column (GE Healthcare) connected to an ÄKTA Start system (GE Healthcare) equilibrated with 50 mM Tris/HCl pH 8.0 and 0.3 M NaCl (buffer-A). *An*AA16A was eluted using a linear gradient from 0 to 100% of 1 M imidazole within 10 column volumes. The fractions containing the purified enzyme were pooled and concentrated using Amicon Ultra 15 mL centrifugal filters (Merck Millipore) with a cutoff of 10 kDa. The *C*-terminal His-tag cleavage and removal with tobacco etch virus (TEV) protease was performed according to Kadowaki et al.^33^ The nontagged *An*AA16A was then saturated with copper by incubating the protein solution with a three-fold molar excess of Cu(II)SO_4_ for 10 min at room temperature, followed by size-exclusion chromatography on a pre-equilibrated HiLoad 16/60 Sephadex 75 size-exclusion column (GE Healthcare) in 50 mM Tris/HCl buffer pH 8.0 containing 150 mM NaCl. Protein purity was analyzed by SDS-PAGE using Coomassie Brilliant Blue G-250 staining (Sigma-Aldrich) and the concentration was determined using the Bradford method using bovine serum albumin as a standard.

### 2.3 Expression, Production, and Purification of *Mt*LPMO9s and *Nc*LPMO9s

Six well-characterized AA9 LPMOs were used in this study (Table 1). *Mt*LPMO9E, *Mt*LPMO9H, and *Mt*LPMO9I were homologously expressed in a low protease/low (hemi)cellulase-producing *M. thermophila* C1 strain^30,31^ and purified as described elsewhere.^32,34^ The expression and purification of *N. crassa* LPMOs produced in *P. pastoris* X-33 (*Nc*LPMO9C, *Nc*LPMO9F, and *Nc*LPMO9M) have been described previously.^13,35^*Mt*LPMO9s and *Nc*LPMO9s were Cu(II)-saturated during their production, and thus, no extra Cu(II) saturation step was performed.

**Table 1:** AA9 LPMOs from *M. thermophila* and *N. crassa* Used in This Study and Corresponding References (Refs)

LPMO	gene name	UniProt ID	CBM	regioselectivity	refs
*M. thermophila*, Produced in *M. thermophila* C1					
*Mt*LPMO9B	MYCTH_80312	G2QCJ3	CBM1	C1	(12)
*Mt*LPMO9E	MYCTH_79765	G2Q7A5	no	C4	(32)
*Mt*LPMO9H	MYCTH_46583	G2Q9T3	CBM1	C1/C4	(36)
*Mt*LPMO9I	MTCTH_2299721	G2Q774	no	C1	(32)
*N. crassa*, Produced in *P. pastoris* X-33					
*Nc*LPMO9C	NCU02916	Q7SHI8	CBM1	C4	(13, 35)
*Nc*LPMO9F	NCU03328	Q873G1	no	C1	
*Nc*LPMO9M	NCU07898	Q7SA19	no	C1/C4	

### 2.4 Cu(II) Saturation of *Mt*AA16A and *An*AA16A

Cu(II) saturation of *Mt*AA16A was performed according to Loose et al.^37^ with modifications. A pure *Mt*AA16A stock solution (1 mg mL^–1^, 500 μL) was incubated with a three-fold molar excess of Cu(II)SO_4_ in 50 mM ammonium acetate pH 5.0 for 30 min at 25 °C under shaking at 600 rpm (Eppendorf ThermoMixer C, Eppendorf, Hamburg, Germany). Excess Cu(II) was removed by a five-cycle washing-out procedure. For each washing step, 500 μL of Cu(II)-saturated *Mt*AA16A was concentrated 10-fold using Amicon Ultra-0.5 centrifugal filters (Sigma-Aldrich) and subsequently brought back to 500 μL by adding 50 mM ammonium acetate pH 5.0. The final concentration of excess Cu(II) was calculated lower than 0.7 pM. In this study, *Mt*AA16A represents the Cu(II)-saturated form, unless mentioned otherwise. For Cu(II) saturation of *An*AA16A, see the previous section. The control sample was prepared in the same way as described above but without *Mt*AA16A, and it is referred to as only Cu(II) sample.

### 2.5 Determination of H_2_O_2_ Production by the Amplex Red/Horseradish Peroxidase Assay

The method for determining H_2_O_2_ production was based on a previously reported protocol^13^ and performed using a commercial Amplex Red Hydrogen Peroxide/Horseradish Peroxidase (HRP) Assay Kit (catalog number: A22188, Thermo Fisher Scientific, Waltham, Massachusetts). The assay was performed in 96-well plates and followed the manufacturer protocol. Each well contained 50 μL of sample including 1 μM AA9 LPMOs with and without 1 μM *Mt*AA16A in the presence of 1 mM Asc in 50 mM ammonium acetate buffer (pH 5.0). Controls were only buffer, boiled *Mt*AA16A, and only Cu(II) sample (described in Section 2.4), all in the presence of 1 mM Asc. In addition, different concentrations of *An*GOX (10, 1, 0.1, and 0.01 μg mL^–1^) in the presence of 15 mM glucose and 1 mM Asc were also prepared. All samples were mixed with Amplex Red/HRP working reagents (final concentration was 50 μM Amplex Red reagent, 0.1 U mL^–1^ HRP, and 50 mM sodium phosphate pH 7.4) in a total volume of 100 μL, after which the measurement was immediately started in a spectrophotometer at 30 °C. The reactions were performed in triplicate. The Amplex Red reaction product resorufin was determined by measuring the absorbance at 560 nm every 10 min (5 s shaking prior to each measurement) till 360 min. The slope of the initial linear increase in absorption was used for the calculation of the H_2_O_2_-producing activity.^13^ According to the manufacturer, the path length of a 100 μL solution in the 96-well plate is roughly 0.33 cm. An extinction coefficient of resorufin, ε_560_ = 58 mM^–1^ cm^–1^, was used to calculate the H_2_O_2_ concentration. One unit of enzyme activity (U) is defined as the amount of enzyme that catalyzes the production of 1 μmol H_2_O_2_ per min under the assay conditions.

### 2.6 Incubations of AA9 LPMOs and AA16 Enzymes with RAC

General incubation settings were 50 mM ammonium acetate buffer (pH 5.0), 2 mg mL^–1^ RAC, and 1 μM AA9 LPMO and/or 1 μM AA16 enzyme. The incubation has been performed in five ways:

(i) Incubations (reaction volume of 300 μL each) containing 1 μM *Mt*LPMO9B with and without 1 μM *Mt*AA16A or *An*AA16A were done in the presence of 1 mM Pyg at 30 °C for 16 h.
(ii) To monitor the generation of H_2_O_2_ and oxidized cello-oligosaccharides over time, incubations (reaction volume of 1200 μL each) containing 1 μM *Mt*LPMO9B with and without 1 μM *Mt*AA16A or *An*AA16A were performed in the presence of 1 mM Pyg, and 300 μL of sample was taken at 2, 4, and 6 h.
(iii) Incubations with H_2_O_2_ (reaction volume of 600 μL each) were initiated by adding 12 μL aliquots of different H_2_O_2_ stock solutions (0, 500, 1250, 2500, 5000, and 10 000 μM) to reach H_2_O_2_ concentrations of 0, 10, 25, 50, 100, and 200 μM in the presence of 1 mM Asc and 1 μM *Mt*LPMO9B or *Nc*LPMO9M. Every 1 h, 12 μL of the different H_2_O_2_ stock solutions were added to the incubations (six additions in total in the first 5 h). Two more samples containing 1 μM *Mt*LPMO9B and 1 μM *Mt*AA16A or *An*AA16A in the presence of 1 mM Asc and no H_2_O_2_ were included as well. The final reaction volume in these two incubations was adjusted (by adding water) to give the same enzyme concentrations as in the incubations with H_2_O_2_ addition. At 6 h, 300 μL of sample was taken out from each incubation, and the remaining solutions were incubated for another 10 h.
(iv) Incubations (reaction volume of 300 μL each) with 1 μM *Mt*LPMO9E, *Mt*LPMO9I, *Mt*LPMO9H, *Nc*LPMO9C, *Nc*LPMO9M, or *Nc*LPMO9F with and without 1 μM *Mt*AA16A or *An*AA16A in the presence of 1 mM or 50 μM Asc were performed at 30 °C for 6 and 16 h.
(v) Incubations (reaction volume of 1200 μL each) with 1 μM *Mt*LPMO9B or *Nc*LPMO9C with and without 1 μM *Mt*AA16A in the presence of 1 mM Asc were performed at 30 °C. Controls were *Mt*LPMO9B or *Nc*LPMO9C with 1 μM boiled *Mt*AA16A (boiled at 95 °C for 20 min) and only Cu(II) sample (described in Section 2.4). Another set of incubations with 1 μM *Mt*LPMO9B or *Nc*LPMO9C with 0.12 μg mL^–1^*An*GOX in the presence of 1 mM Asc and 15 mM glucose were carried out at 30 °C. At 1, 2, 3, 4, 5, 6, and 16 h of incubation, a 200 μL sample of each reaction was collected.

All supernatants from the above incubations were collected and stored at −20 °C for further analysis. All incubations were performed in duplicate.

### 2.7 Determination of H_2_O_2_ by the Ferric-Xylenol Orange Assay

The level of H_2_O_2_ in the supernatants after 2, 4, and 6 h incubation of *Mt*LPMO9B with/without *Mt*AA16A and *An*AA16A in the presence or absence of RAC and Pyg was determined using the Peroxide Assay Kit (catalog number: MAK311, Sigma-Aldrich). The assay was performed by following the protocol provided by the manufacturer. First, H_2_O_2_ standards (0, 3, 6, 9, 12, 18, 24, and 30 μM) and detection reagent (mixing 1 volume of reagent A with 100 volumes of reagent B) were freshly prepared. Afterwards, 40 μL of undiluted supernatants from the incubations and H_2_O_2_ standards were added into separate wells of a 96-well plate. Subsequently, 200 μL of detection reagent was added into wells, and the reactions were incubated for 30 min at room temperature. The absorbance of each sample at 585 nm was determined in a spectrophotometer. The H_2_O_2_ levels were calculated based on a calibration curve generated by H_2_O_2_ standards. All measurements were performed in duplicate.

### 2.8 HPAEC-PAD Analysis for Oligosaccharide Profiling and Relative Quantification of Products

All supernatants from the incubations of AA9 LPMO (in the presence and absence of AA16s) with RAC were analyzed by HPAEC. The analysis was performed on an ICS-5000 system (Dionex, Sunnyvale, California) equipped with a CarboPac PA-1 column (2 mm ID × 250 mm; Dionex) in combination with a CarboPac PA guard column (2 mm ID × 50 mm; Dionex). The system was further equipped with pulsed amperometric detection (PAD). Mobile phases were (A) 0.1 M NaOH and (B) 1 M NaOAc in 0.1 M NaOH. The column temperature was set at 20 °C. The elution profile applied has previously been described.^12,32^ Samples were diluted five times before analysis. For supernatants collected in Section 2.6 (v), the total peak area of released oxidized cello-oligosaccharides was calculated.

### 2.9 Crystallization, Structure Determination, and Structure Modeling

Prior to crystallization, *Mt*AA16A was treated with endoglycosidase H (Sigma-Aldrich) according to Frandsen et al.^38^ with modifications. In brief, a 1 mL *Mt*AA16A (10 mg) solution in 50 mM NaOAc pH 6.0 containing 150 mM NaCl was incubated with 100 μL endoglycosidase H (0.5 U based on the manufactural information) for 16 h at room temperature. Afterward, the incubated sample was exchanged to 20 mM NaOAc buffer pH 5.5. Crystallization was set up with a protein stock solution of 20 mg mL^–1^ preincubated in sample buffer for at least 1 h with equimolar Cu(II) acetate. Crystallization trials with commercial screens JCSG+ (Qiagen, Hilden, Germany), Index (Hampton Research, Aliso Viejo, California), PEG/Ion (Hampton Research), and Morpheus (Molecular Dimensions, Sheffield, U.K.) were set up with an Oryx-8 crystallization robot (Douglas Instruments, Hungerford, U.K.) using the sitting drop vapor diffusion method in MRC-2-drop 96-well plates at room temperature. The drops had a volume of 0.3 μL consisting of protein stock solution to reservoir in ratios of 3:1 and 1:1. Diffracting crystals/needles were obtained in different conditions, and data were collected at 100 K without additional cryoprotection. Crystals grown from the JCSG+ screen (0.2 M CaOAc, 0.1 M Na–cacodylate pH 6.5, and 40% v/v PEG 300) diffracted well but did not lead to structure determination due to possible twinning. Crystals grown from the Morpheus screen (0.1 M buffer system 1 (pH = 6.5), 30% EDO_P8K, and 0.09 M halogens)^39^ led to a preliminary structure determination at 3.1 Å resolution. Optimization of similar Morpheus conditions in MRC MAXI 48-well plates with 1 μL of protein stock and 1 μL of reservoir (prepared by diluting the Morpheus condition with water: 0.1 M buffer system 1, 30% EDO_P8K, divalent cations, 300 μL in a ratio of 9:1) led to a good data set (Table S1). Diffraction tests and collections were carried out at the ID30A-3 beamline^40^ at ESRF (Grenoble, France) and BioMAX^41^ beamline at MAX IV (Lund, Sweden), and data was processed both through the available automatic pipelines and manually using XDSAPP software^42^ of the PReSTO platform or XDS.^43^ Molecular replacement was carried out in MOLREP^44^ with an AlphaFold 2^45,46^ model of *Mt*AA16A obtained through the Colab implementation.^47^ A clear solution with three molecules in the asymmetric unit was obtained, which was further refined with REFMAC5^48^ and consecutive manual model building by COOT,^49−51^ yielding a good quality structure with a maximum resolution of 2.65 Å. Crystallographic statistics are given in Table S1. The structure has been deposited in the Protein Data Bank (PDB) with the accession number 7ZE9. Figures were rendered in PyMOL (v2.0.1 2018, Schrödinger, Inc., New York).

## 3 Results and Discussion

### 3.1 *Mt*AA16A: Molecular Mass, *N*-Glycosylation, and Methylation of *N*-Terminal Histidine

Purified *Mt*AA16A showed a major band at 27 kDa in SDS-PAGE (Figure S1). Since the predicted molecular mass of *Mt*AA16A based on the amino acid sequence without a signal peptide is 18.4 kDa (Figure S2), glycosylation of *Mt*AA16A was expected, as also observed with other homologously produced *Mt*LPMO9s.^12^ Indeed, after treatment of *Mt*AA16A with (*N*-acetyl-β-glucosaminyl)asparagine amidase (PNGase F), a major band at 19 kDa (Figure S1) remained, indicating that *Mt*AA16A contained *N*-glycosylation. The predicted molecular mass of *An*AA16A is 19.9 kDa (catalytic domain). Similar to *Mt*AA16A, in the SDS-PAGE experiment, a roughly 30 kDa band was visible, indicative of glycosylation (Figure S1).

Typical for homologously expressed fungal LPMOs is the methylated *N*-terminal histidine, of which the methylation is suggested to play a role in protection against auto-oxidation of the copper histidine brace active site.^52^ Reversed phase liquid chromatography coupled to mass spectrometry (LC-MS/MS*^n^*) of a tryptic digest revealed that the *N*-terminal histidine of *Mt*AA16A was indeed methylated (MeHis1; Figure S3). Further identification of peptides in the *Mt*AA16A tryptic digest confirmed that the amino acid sequence of the *Mt*AA16A protein was in accordance with the prediction based on gene annotation.

The amino acid sequence of *An*AA16A was also confirmed, as well as the expected nonmethylated *N*-terminal histidine (data not shown).

### 3.2 *Mt*AA16A Does Not Oxidatively Cleave Carbohydrates but Oxidizes Syringol-like Compounds

The AA16 family has been suggested to comprise catalytic LPMO-like enzymes, though this suggestion was based on a rather low C1-oxidative cleavage of cellulose observed for only one AA16 candidate (*Aa*AA16).^11^*Mt*AA16A did not oxidatively cleave cellulose, and none of the other carbohydrates were tested, including cellopentaose, cellohexaose, chitin, pectin, hemicelluloses, and combinations thereof (Table S2). Oxidative cleavage was neither observed after renewing Cu(II) saturation of *Mt*AA16A nor by varying the type of the reducing agent or adding H_2_O_2_ and also not by increasing substrate or enzyme concentrations (Table S2). We also observed that no oxidized products were released by *An*AA16A from cellulosic substates including phosphoric acid swollen cellulose, Avicel PH-101, and cellulose nanocrystals (data not shown). Therefore, we concluded that *Mt*AA16A and *An*AA16A have no typical LPMO-like catalytic action toward cellulose and other investigated poly- and oligosaccharides.

Next, we questioned whether *Mt*AA16A and *An*AA16A actually are oxidative enzymes or just noncatalytic copper-containing proteins, similar to Bim1^8^ or *La*X325.^9^ Therefore, we tested if *Mt*AA16A was active in the H_2_O_2_-driven conversion of 2,6-dimethoxyphenol (syringol; Syr).^53^*Mt*LPMO9B, *Mt*LPMO9E, *Mt*LPMO9H, and *Mt*LPMO9I served as the reference, and results are shown in Figure S4. Following the formation of the chromogenic product coerulignone, it allowed the estimation of the specific activity of all *Mt*LPMO9s to range between 0.27 and 0.56 U g^–1^. However, *Mt*AA16A showed a much higher specific activity of 4.14 U g^–1^. These results indicated that *Mt*AA16A is a copper-dependent enzyme able to oxidize syringol in the presence of H_2_O_2_.

### 3.3 *Mt*AA16A Crystal Structure

**Figure 2:**
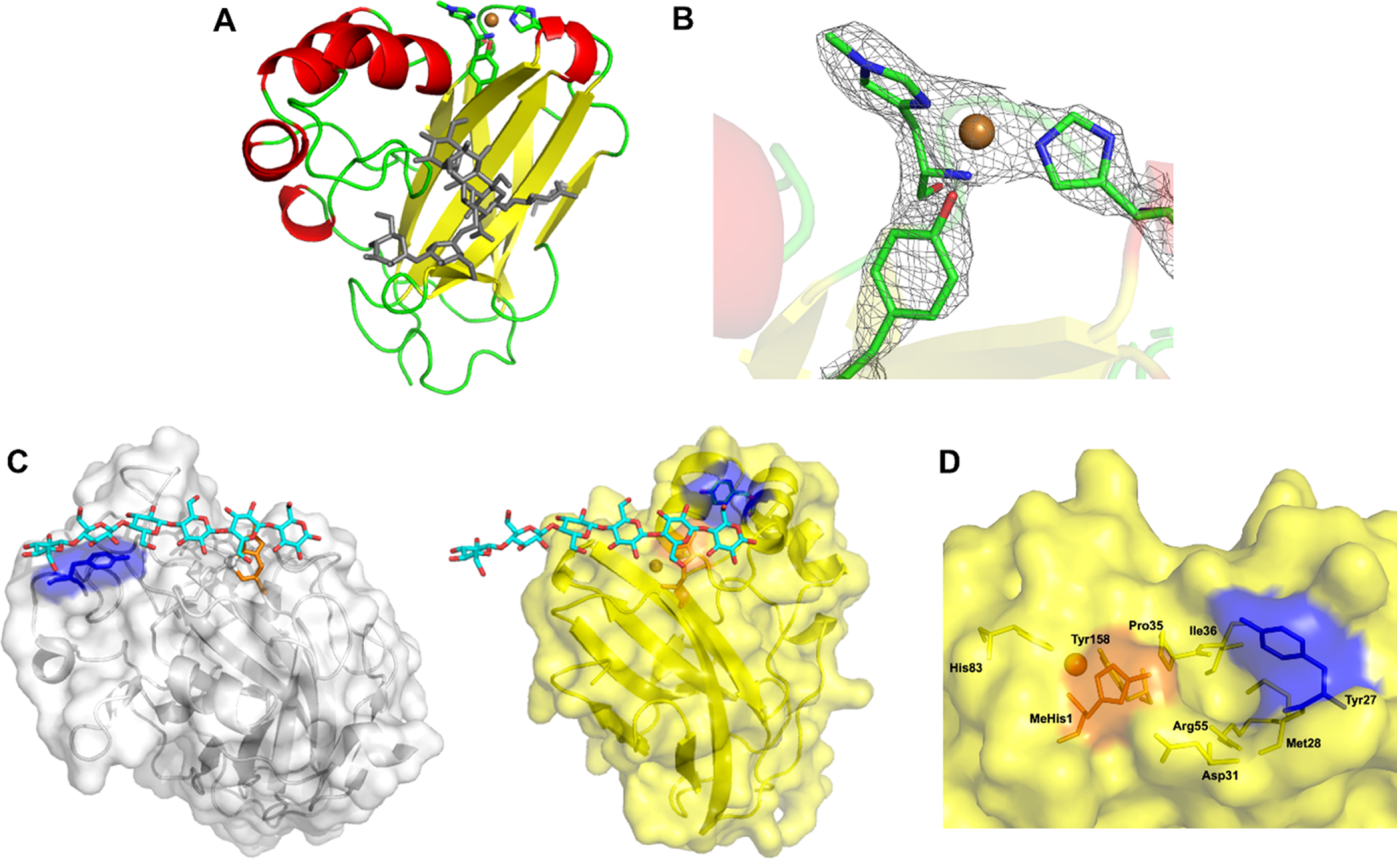
(A) Overall cartoon representation of *Mt*AA16A showing the different secondary structure elements and the copper (orange sphere) binding site in stick representation. (B) Close-up of the copper binding site, including the 2*F*_obs_ – *F*_calc_ density map at the 1.0 σ level. (C) Side-by-side surface views of *Ls*AA9A (in white) with bound cellohexaose (PDB entry: 5ACI) and *Mt*AA16A (in yellow with cellohexaose overlayed from the 5ACI structure). (D) Close-up of the surface near Tyr27. His1 is in orange, and Tyr residues on the presumed substrate-binding surface are in blue. Note that the aromatic ring of Tyr27 in *Mt*AA16A lies perpendicular rather than parallel to the protein surface and is adjacent to a small pocket which could perhaps accommodate small molecules like syringol.

To better understand why both AA16s did not catalyze the oxidative cleavage of poly- and oligosaccharides, we determined the crystal structure of *Mt*AA16A. The three-dimensional structure of *Mt*AA16A, the first experimental structure in the AA16 family, shows the typical LPMO fold (Figure 2A).^54^ A search with DALI^55,56^ revealed high structural similarity with AA9, AA10, and AA11 family members, but *Mt*AA16A has a significantly smaller size than the matched structures (see Figure S5 for comparison). The copper binding site appears to be identical to the one observed in AA9, AA11, AA13, AA15, AA17, and some AA10 members with the His-brace providing three equatorial ligands and an additional Tyr axial ligand (Figures 2B, S6, and Table S3) with typical distances from the copper to the N ligands (1.8–2.3 Å) and a longer distance to the Tyr–OH (2.7 Å). The methylation of His1 is confirmed in the structure. In the crystal, a carboxylic residue from a neighboring molecule blocks the equatorial position to the copper, expecting to bind water in solution. There is no visible axial ligand, presumably due to photoreduction of the Cu(II) to Cu(I) under X-ray exposure.^38^ The θ angles in Table S3 are also consistent with a Cu(I) state. Generally, the geometry of the Cu site looks fully compatible with reactivity, which further supports the demonstrated oxidizing activity on small compounds such as syringol. Second coordination sphere residues include a Gln, which is in the same position as an important and conserved Gln in AA9 (Figure S6),^57^ while the conserved second coordination sphere His from AA9 is substituted by an Asn. Trp149 is able to make π–π stacking interactions with the active site residue Tyr158, which is reminiscent of similar interactions in AA10 and AA11 family members (Figure S6).^58^

Interestingly, the longest molecular axis in AA16 runs in a different direction from the longest axis in the closest DALI hits or *Ls*AA9A,^38^ an AA9 LPMO for which information on cello-oligosaccharide binding is available (Figure 2C). The surface adjacent to the His-brace in *Mt*AA16A lacks the typical flat aromatic features seen in most cellulose-binding LPMOs and exemplified in the complex of *Ls*AA9A with cellohexaose by interaction with a Tyr. At the surface of *Mt*AA16A, another Tyr residue (Tyr27) can be found near the His-brace but lies sideways rather than parallel to the protein surface. Thus, *Mt*AA16A does not seem to possess a likely polysaccharide-binding surface adjacent to the His-brace but rather a small pocket (Figure 2D), which could be speculated to interact with small aromatics like syringol, on which we have demonstrated activity. Binding experiments (Figure S7) indicated that, as suggested by the crystal structure, *Mt*AA16A was not able to bind to RAC. In addition, thermal shift assays supported that *Mt*AA16A did not bind to cello-oligosaccharides (DP2–6), while syringol induced significant thermal stabilization (Figure S8), which is consistent with binding. However, as this is not a direct binding assay, an alternative possibility could be that the reduction of the active site metal results in stabilization. Distant from the putative substrate-binding surface, *N*-glycosylation at Asn89 is very obvious in the electron density, and despite treatment with endoglycosidase H, 5–7 glycan units are visible at Asn89, interacting with exposed Phe52, Asn98, and Tyr148. One NAG unit is also visible at Asn126.

### 3.4 AA16 Enzymes Produce H_2_O_2_ to Boost the *Mt*LPMO9B-Driven Oxidative Cleavage of Cellulose

**Figure 3:**
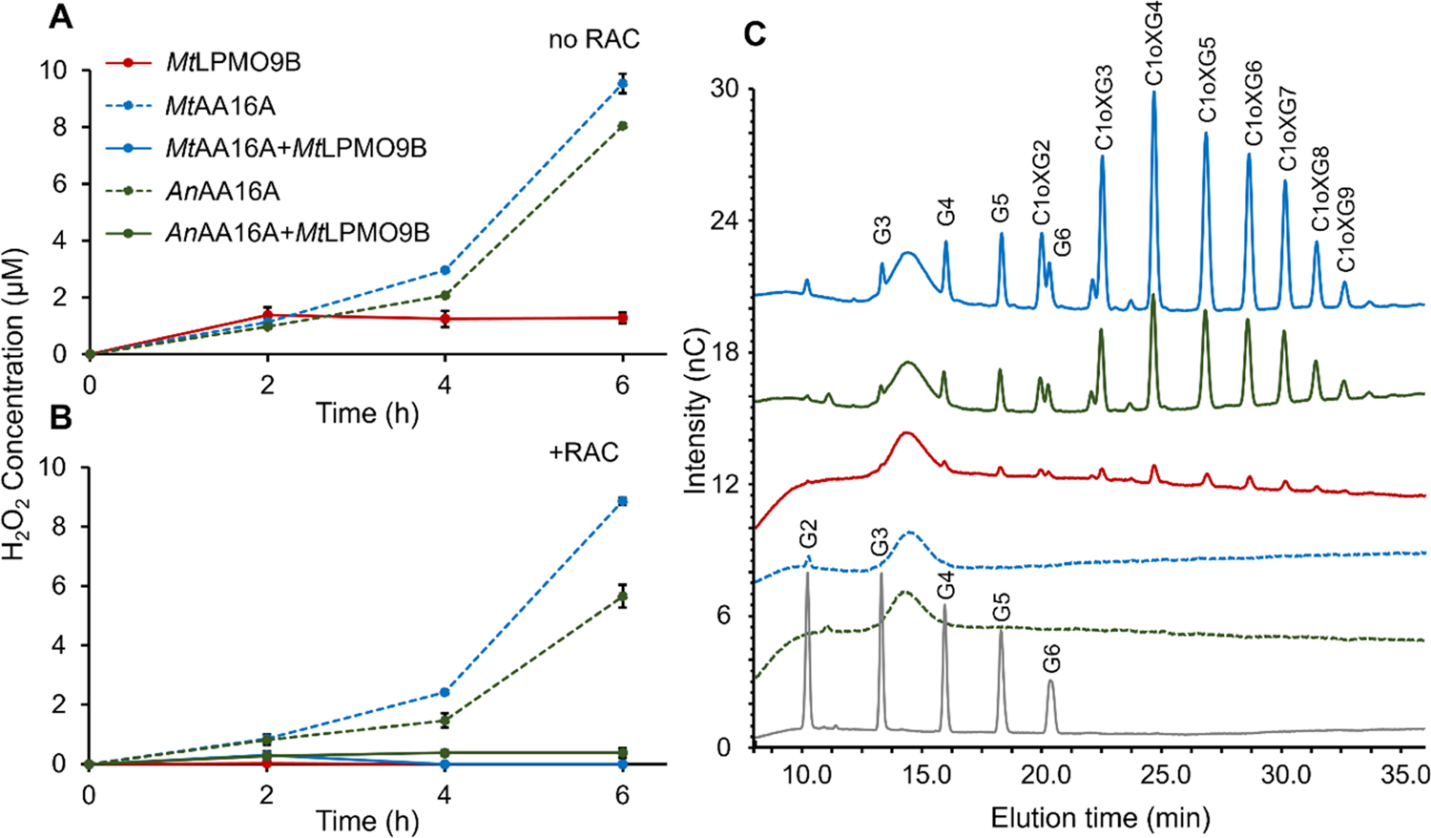
(A) H_2_O_2_ concentration in the presence of Pyg and absence of RAC over time. (B) H_2_O_2_ concentration in the presence of both Pyg and RAC over time. (C) Oligosaccharide elution patterns determined by HPAEC. RAC samples were incubated for 16 h with only *Mt*LPMO9B (red line), only *An*AA16A (green dotted line), only *Mt*AA16A (blue dotted line), *An*AA16A + *Mt*LPMO9B (green line), and *Mt*AA16A + *Mt*LPMO9B (blue line) in the presence of Pyg. The signal intensity of each peak and elution profiles in duplicated incubations are comparable, and only one chromatogram is shown here. Standards of cello-oligosaccharides (DP2–6 (G2–G6), 1 μg mL^–1^ each) are shown in gray. Annotation of nonoxidized (G2–G6) and C1-oxidized cello-oligosaccharides (C1oxG2–C1oxG9) is based on a previous study.^32^ HPAEC chromatograms of control samples and other time points (2, 4, and 6 h) are shown in Figures S9 and S10.

We further questioned whether *Mt*AA16A and *An*AA16A display oxidase activity, as earlier reported for *Aa*AA16 (Figure 1, route 3).^8^ Indeed, we observed accumulation of H_2_O_2_ in *Mt*AA16A-Pyg and *An*AA16A-Pyg samples, while accumulation of H_2_O_2_ in *Mt*LPMO9B-Pyg samples was absent in the presence of RAC or very low in the absence of RAC (Figure 3). Accumulation of H_2_O_2_ was also absent or lower than 1 μM in incubations with *Mt*LPMO9E, *Mt*LPMO9H, and *Mt*LPMO9I without RAC (data not shown). Apparently, *Mt*LPMO9s are poor H_2_O_2_ producers in contrast to *Nc*LPMO9s (Table 2). H_2_O_2_ production rates were further assessed and are discussed in Section 3.5. No H_2_O_2_ accumulation occurred in the RAC sample in the presence of Pyg when *Mt*LPMO9B was combined with either *Mt*AA16A or *An*AA16A (Figure 3).

**Table 2:** H_2_O_2_-Producing Activity of *Mt*AA16A, *Mt*LPMO9s, and *Nc*LPMO9s in the Presence of 1 mM Asc^a^

H_2_O_2_-producing activity (mU)^b^	
Cu(II) only	2.9 ± 1.3
boiled *Mt*AA16A	5.0 ± 1.7
*Mt*AA16A	74.2 ± 3.3
*Mt*LPMO9B	16.6 ± 0.5
*Mt*LPMO9E	3.2 ± 0.5
*Mt*LPMO9H	45.4 ± 7.0
*Mt*LPMO9I	41.3 ± 0.8
*Nc*LPMO9C	301.9 ± 2.6
*Nc*LPMO9F	32.8 ± 0.8
*Nc*LPMO9M	510.0 ± 9.6

a H_2_O_2_-producing activity of *Mt*AA16A, *Mt*LPMO9s, and *Nc*LPMO9s in the presence of 50 μM Asc is shown in Table S4.

b See the Experimental Section for assay conditions.

Based on the above observations and the reported oxidase activity of LPMOs,^16^ we hypothesized that *Mt*AA16A produces H_2_O_2_ to drive the peroxygenase reaction of *Mt*LPMO9s in cleaving RAC (Figure 1, route 1). Therefore, we incubated both *Mt*LPMO9B and *Mt*AA16A and a mixture of these enzymes, with RAC in the presence or absence of Pyg, and reactions were analyzed by HPAEC-PAD (Figure 3). Both *Mt*-enzymes were free of hydrolytic side activity and, as mentioned in the previous section, *Mt*AA16A did not release (oxidized) oligosaccharides from RAC (Figures 3 and S9). Interestingly, in the combined *Mt*AA16A and *Mt*LPMO9B-RAC incubation (Figure 3), a pronounced higher amount of nonoxidized (Glc) and oxidized cello-oligosaccharides (GlcOx) was released than in the same incubation with *Mt*LPMO9B alone. Likewise, when the *Mt*LPMO9B-RAC reaction was performed in the presence of *An*AA16A, the increase in released products was substantial (Figure 3).

These findings provided support for our hypothesis that, in the presence of a reducing agent, *Mt*AA16A and *An*AA16A produce H_2_O_2_ that can act as a cosubstrate for *Mt*LPMO9B peroxygenase reactions in cleaving RAC. A comparable scenario has been described by Stepnov and co-workers, who observed that H_2_O_2_ was continuously produced *in situ* by a CBM-truncated *Sc*LPMO10C_TR_ (only catalytic domain) to boost the full-length *Sc*LPMO10C in degrading cellulose.^28^

### 3.5 AA16 Enzymes Boost Other *Mt*LPMO9s but Not *Nc*LPMO9s

**Figure 4:**
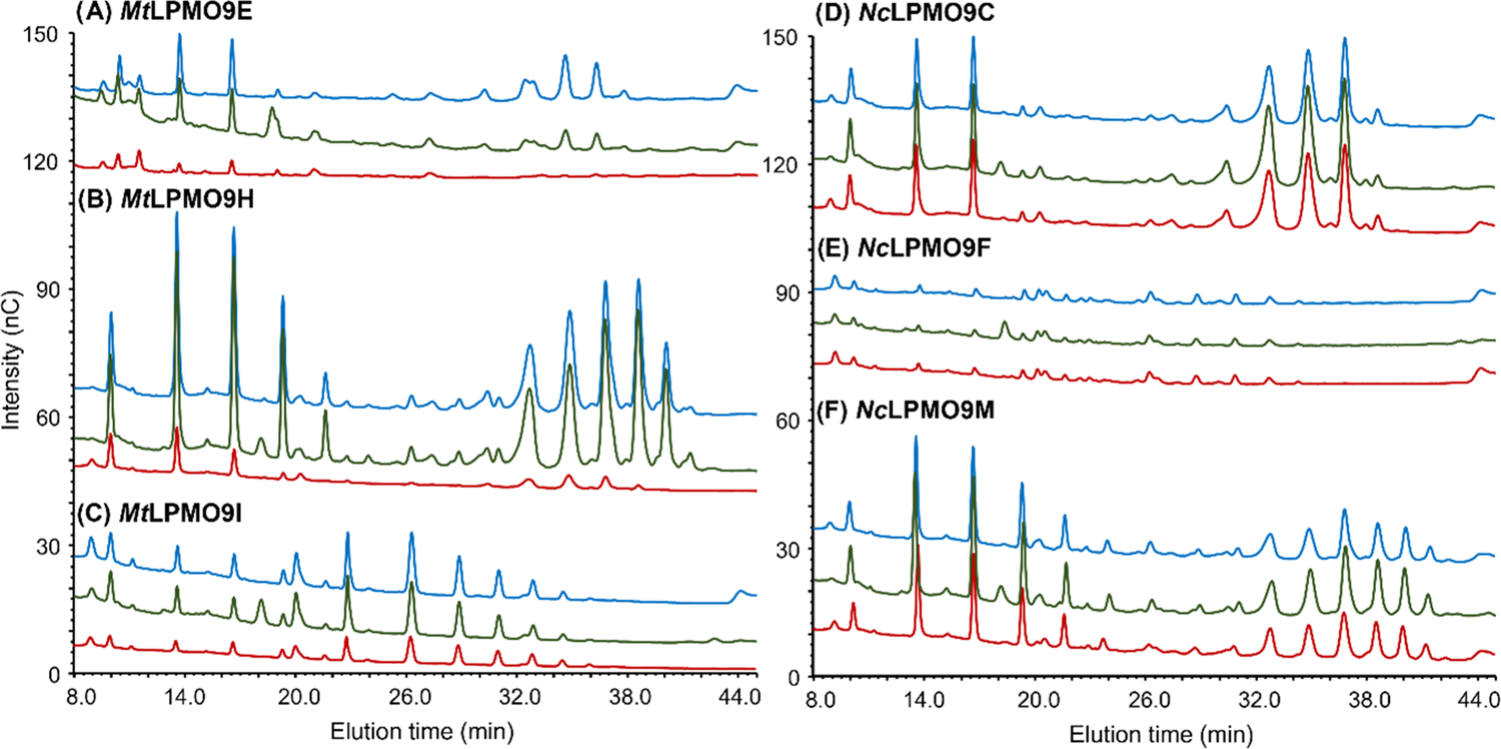
HPAEC chromatograms of RAC samples incubated with various AA9 LPMOs ((A) *Mt*LPMO9E, (B) *Mt*LPMO9H, (C) *Mt*LPMO9I, (D) *Nc*LPMO9C, (E) *Nc*LPMO9F, and (F) *Nc*LPMO9M) in the presence of 1 mM Asc after 16 h. HPAEC chromatograms of these incubations at 6 h are shown in Figure S11. RAC samples incubated for 16 h with only LPMO, LPMO + *An*AA16A, and LPMO + *Mt*AA16A are shown in red, green, and blue lines, respectively. The signal intensity of each peak and elution profiles in duplicated incubations are comparable, and only one chromatogram is shown here.

Apart from *Mt*LPMO9B, *Mt*LPMO9E, *Mt*LPMO9H, and *Mt*LPMO9I were also boosted by the AA16s in oxidatively degrading cellulose (Figure 4A–C; 16 h incubations). Intriguingly, the situation was different for combinations of the AA16s with *Nc*LPMO9C, *Nc*LPMO9F, and *Nc*LPMO9M (Figure 4D–F; 16 h incubations). A shorter incubation (6 h) of RAC + Asc with *Nc*LPMO9s also showed no significant increase in oxidized products by AA16 addition (Figure S11).

The different boosting effects seen on *Mt*LPMO9s and *Nc*LPMO9s could be due to the different H_2_O_2_-producing abilities of individual LPMOs, as shown by Kittl and co-workers.^13^ To determine the H_2_O_2_ production rate of *Mt*LPMO9s and *Nc*LPMO9s, the Amplex Red/HRP assay was used instead of the ferric-xylenol orange assay. The former assay measures the H_2_O_2_ level continuously (immediate reaction with H_2_O_2_), while the latter assay determines the steady level of H_2_O_2_ in the sample (after 30 min incubation with the reagent) that could lead to the underestimation of the H_2_O_2_-producing rate.

From Table 2, it follows that *Mt*AA16A had a higher H_2_O_2_-producing activity (74.2 ± 3.3 mU) compared to all four *Mt*LPMO9s. H_2_O_2_-producing activities for the boiled *Mt*AA16A and equivalent amount of Cu(II) only samples were 5.0 ± 1.7 and 2.9 ± 1.3 mU, respectively. *Mt*LPMO9s showed a lower H_2_O_2_-producing activity (*Mt*LPMO9B, 16.6 ± 0.5 mU; *Mt*LPMO9E, 3.2 ± 0.5 mU; *Mt*LPMO9H, 45.4 ± 7.0 mU; *Mt*LPMO9I, 41.3 ± 0.8 mU) compared to *Nc*LPMO9C (301.9 ± 2.6 mU) and *Nc*LPMO9M (510.0 ± 9.6 mU). These results indicated that *Mt*LPMO9s are relatively poor H_2_O_2_ producers, and thus, these LPMOs are likely to be boosted by the H_2_O_2_-producing AA16s *in situ*. Differently, *Nc*LPMO9C and *Nc*LPMO9M were able to produce larger amounts of H_2_O_2_, and it seems that they do not need more H_2_O_2_ for their reaction, as substantiated by the absence of boosting by the AA16s (i.e., for *Nc*LPMO9C and *Nc*LPMO9M). *Nc*LPMO9F showed low H_2_O_2_-producing activity (32.8 ± 0.8 mU). However, previous studies showed its rapid inactivation and low catalytic ability,^13,35,53^ which could explain the lack of boosting observed.

We also determined the H_2_O_2_-producing activity of *Mt*AA16A, *Mt*LPMO9s, and *Nc*LPMO9s in the presence of 50 μM Asc (Table S4), the comparable concentration as reported by Kittl and co-workers.^13^ From calibration curves, it turned out that the presence of Asc in the Amplex Red/HRP assay led to the underestimation of H_2_O_2_ levels; however, 50 μM or 1 mM concentrations of Asc gave no differences in the absorbance at 560 nm (Figure S12). In the presence of 50 μM Asc, *Nc*LPMO9C and *Nc*LPMO9M still displayed higher H_2_O_2_-producing activity than *Mt*LPMO9s (Table S4), although all enzyme activities were lower than those in the presence of 1 mM Asc (cf. Table 2).

### 3.6 Stepwise Addition of H_2_O_2_ is Less Effective than AA16 Enzyme Supply to Drive the *Mt*LPMO9B Peroxygenase Reaction

**Figure 5:**
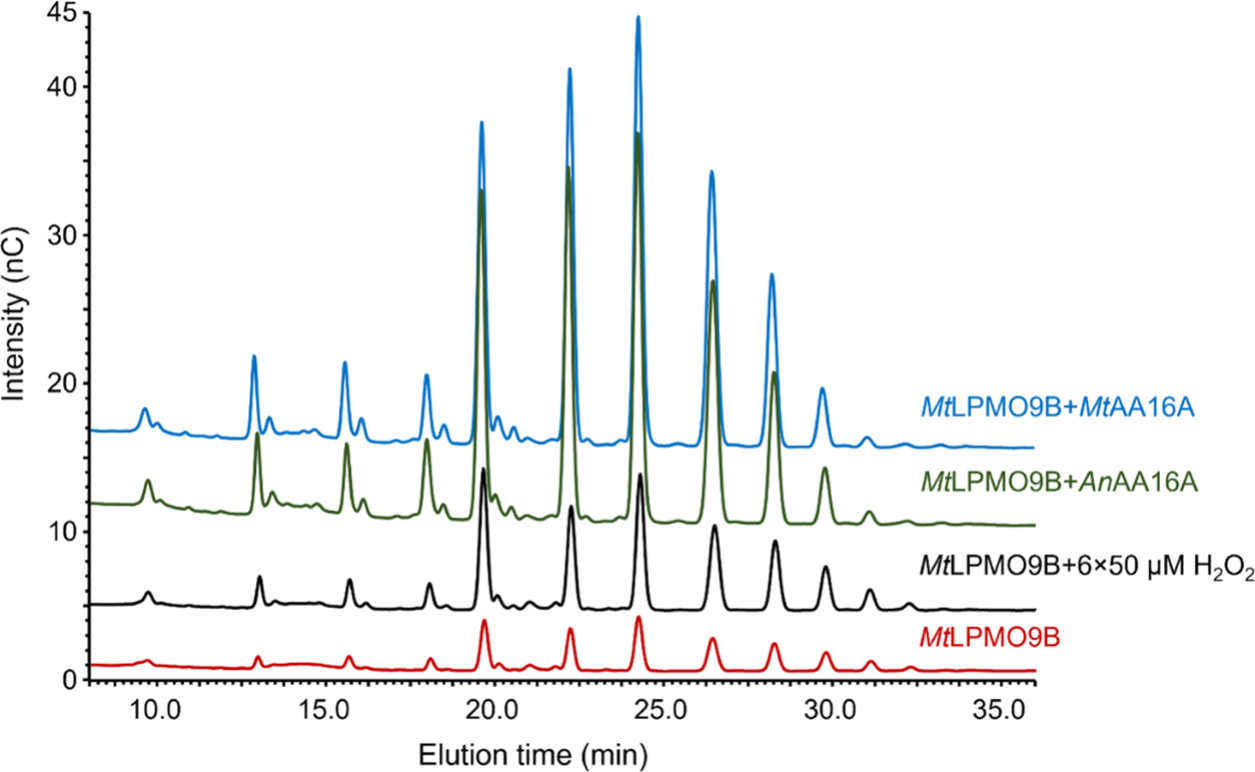
HPAEC elution patterns of RAC samples incubated with only *Mt*LPMO9B (red line), *Mt*LPMO9B with the addition of 50 μM H_2_O_2_ (in total six times) (black line), *Mt*LPMO9B + *An*AA16A (green line), and *Mt*LPMO9B + *Mt*AA16A (blue line) in the presence of Asc at 16 h. HPAEC chromatograms of control samples are shown in Figure S15. Compared to all concentrations of H_2_O_2_, the highest activity was found when adding 50 μM H_2_O_2_ to *Mt*LPMO9B-RAC digest at 6 and 16 h. HPAEC chromatograms of *Mt*LPMO9B with the addition of 0, 10, 25, 100, and 200 μM H_2_O_2_ (in total six times) in the presence of Asc are shown in Figure S14. The signal intensity of each peak and elution profiles in duplicated incubations are comparable, and only one chromatogram is shown here.

Manual stepwise addition of H_2_O_2_ to stimulate the catalytic action of AA9 LPMOs to oxidatively cleave cellulose has been shown effective in other studies.^59−61^ However, this has not been tested for *Mt*LPMO9s. Hence, we compared such a setup with the AA16 supply for *Mt*LPMO9B-RAC-Asc incubations. H_2_O_2_ was added in six equal aliquots at six successive time points during the incubation, versus a single addition of AA16 at the start. *Mt*LPMO9B (+Asc) released oxidized products from RAC (6 h; Figure 5), and the amount of oxidized products was further increased at 16 h (Figure S13). Based on the increased amounts of oxidized products formed at 6 h (Figures 5 and S14), it is concluded that the stepwise addition of H_2_O_2_ (0, 10, 25, 50, 100, and 200 μM) boosted the *Mt*LPMO9B action as expected. For the stepwise addition of 50 μM or higher concentration of H_2_O_2_, no additional oxidized products were formed (Figure S14) after 6 h of incubation, which can be the result of a damaged active site of the *Mt*LPMO9B.^16,23,24^ Notably, the addition of either 1 μM *Mt*AA16A or 1 μM *An*AA16A to 1 μM *Mt*LPMO9B resulted in approximately a three times higher amount of oxidized products (control reactions are shown in Figure S15) compared to the most optimal H_2_O_2_ concentration (50 μM) supplied to the *Mt*LPMO9B-RAC digest (Figure 5).

To test if H_2_O_2_ addition boosted the activity of *Nc*LPMO9s in our experimental setups, H_2_O_2_ (six times 0, 10, 25, 50, 100, and 200 μM) was added to *Nc*LPMO9M-RAC (+Asc) digestions. We observed that oxidative cleavage of RAC by *Nc*LPMO9M was visibly boosted by 10 μM H_2_O_2_ per addition (60 μM in total) (Figures S16 and S17). In a study by Petrović and co-workers, *Nc*LPMO9A, *Nc*LPMO9C, and *Nc*LPMO9D were shown to release considerably increased amounts of oxidized products at 4 h after manual stepwise addition of 45 μM H_2_O_2_.^61^

As described in the previous section, it was anticipated that *Nc*LPMO9s were able to produce sufficient H_2_O_2_ to keep the catalytic activity maximum, but these results demonstrated that there was still room to further increase their activity. Apparently, the fact that AA16s boost *Mt*LPMO9s but not *Nc*LPMO9s cannot be merely explained by the *in situ* H_2_O_2_ production.

### 3.7 Glucose Oxidase Is Less Effective than AA16 Enzymes in Boosting *Mt*LPMO9B Activity

To get more insight into the origin of the boosting effect, we performed time-course incubations of RAC with *Mt*LPMO9B or *Nc*LPMO9C with and without *Mt*AA16A. In addition, we included *An*GOX in the incubations with *Mt*LPMO9B or *Nc*LPMO9C to investigate if the same boosting effect as with AA16s could be achieved. *An*GOX has been shown to produce H_2_O_2_ to drive the peroxygenase reaction of LPMOs.^16^*An*GOX (0.12 μg mL^–1^) was dosed based on the comparable H_2_O_2_-producing activity (76.4 mU) with 1 μM *Mt*AA16A (74.2 ± 3.3 mU) used in the previous experiments. The required *An*GOX concentration was calculated by a calibration curve (activity vs concentration) determined by using different concentrations of *An*GOX (Figure S18).

**Figure 6:**
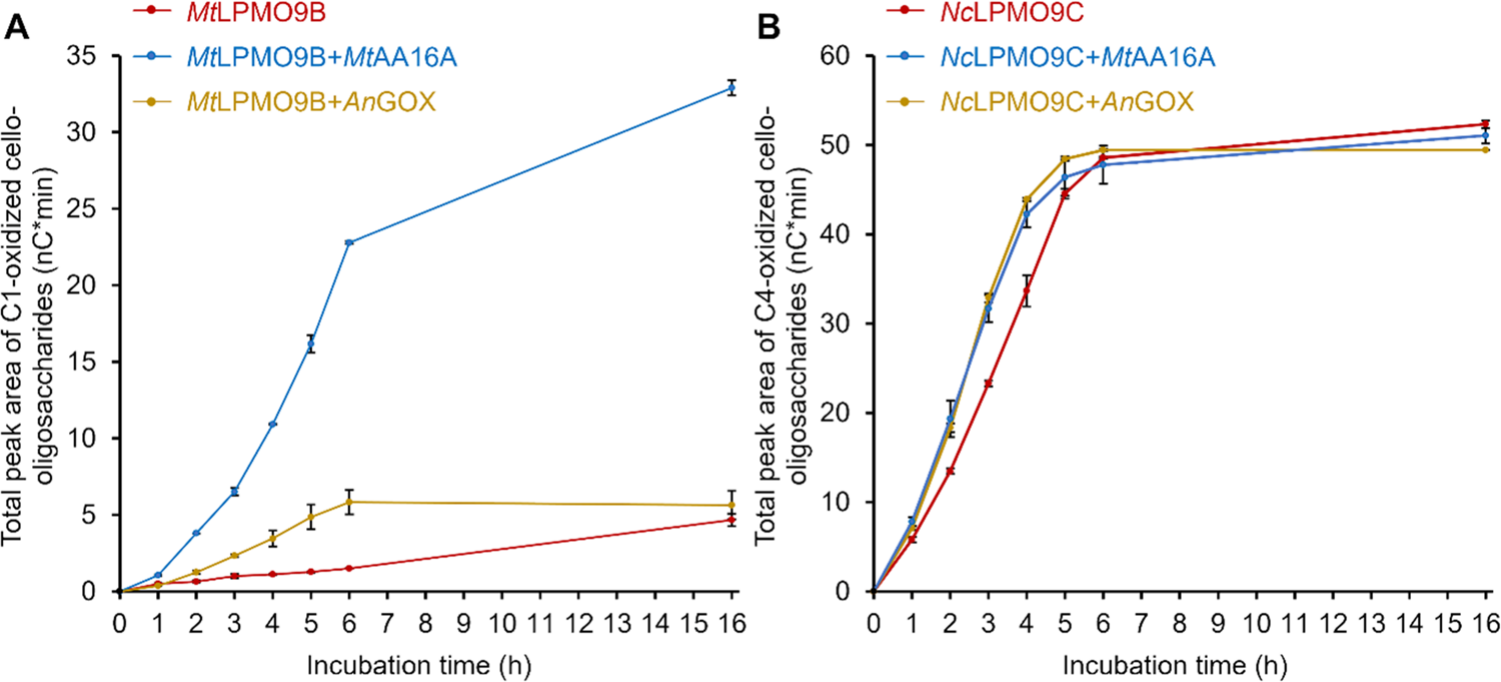
Relative quantification of the total peak area of C1-oxidized cello-oligosaccharides in *Mt*LPMO9B samples (A) or C4-oxidized cello-oligosaccharides in *Nc*LPMO9C samples (B). Red lines are samples with RAC and only *Mt*LPMO9B or *Nc*LPMO9C. Blue lines are samples with RAC and *Mt*LPMO9B (or *Nc*LPMO9C) and *Mt*AA16A. Yellow lines are samples with RAC and *Mt*LPMO9B (or *Nc*LPMO9C) and *An*GOX. The control samples were *Mt*LPMO9B or *Nc*LPMO9C with boiled *Mt*AA16A, where the released oxidized products were the same as for samples with only *Mt*LPMO9B or *Nc*LPMO9C (data not shown). The error bars indicate the standard deviations of duplicate incubations.

As expected from the previous results, *An*GOX boosted *Mt*LPMO9B-RAC degradation till 6 h (Figure 6A). From 6–16 h, *Mt*LPMO9B still released oxidized products from RAC. However, in the presence of *An*GOX, there was no increase of the oxidized product formation by *Mt*LPMO9B after 6 h, indicating that *Mt*LPMO9B was completely inactivated by the H_2_O_2_ produced by *An*GOX. This LPMO inactivation by H_2_O_2_ also has been reported in other studies.^16,23^ Intriguingly, much higher amounts of oxidized cello-oligosaccharides were generated in the *Mt*LPMO9B-RAC sample with *Mt*AA16A compared to the one without *Mt*AA16A, and even approximately 4 times higher than that in the *Mt*LPMO9B-RAC sample with *An*GOX at 6 h. In addition, it was found that the amount of oxidized cello-oligosaccharides was still increasing after 6 h in the *Mt*LPMO9B-RAC sample with *Mt*AA16A, indicating that less inactivation of *Mt*LPMO9B compared to the sample with *An*GOX had occurred (Figure 6A). In other words, *Mt*AA16A boosted *Mt*LPMO9B and somehow also protected *Mt*LPMO9B from the inactivation by H_2_O_2_. These observations strongly indicate, again, that the boosting effect is not only due to the *in situ* H_2_O_2_ production by AA16s.

In contrast to *Mt*LPMO9B-RAC samples, the amount of oxidized products formed in the *Nc*LPMO9C-RAC sample with *Mt*AA16A was only slightly higher than that without *Mt*AA16A, and was equal to the amount in the *Nc*LPMO9C-RAC sample with *An*GOX till 6 h (Figure 6B). From 6–16 h, almost no increase in the formation of oxidized products in all three *Nc*LPMO9C-RAC samples occurred, indicating that *Nc*LPMO9C was inactivated after 6 h.

We attempted to explain the observed different boosting effects on *Mt*LPMO9s and *Nc*LPMO9s by AA16s. We propose the challenging hypothesis that *Mt*AA16A and *An*AA16A interact with *Mt*LPMO9s but not with *Nc*LPMO9s. Such an interaction might assist the transmission of H_2_O_2_ to the catalytic sites of the *Mt*LPMO9s and stimulate their peroxygenase reaction (Figure S19).

Attempts to experimentally confirm the hypothetical protein complex by size-exclusion chromatography and mass spectrometric techniques were not successful. It might be because *Mt*AA16A and *An*AA16A form weak transient interactions with the *Mt*LPMO9s, which is challenging to study as reviewed by Qin et al.^62^ However, of note here is that in a recent study, protein–protein interaction between a cell wall remodeling (CWR) protein CWR-1 AA11 LPMO and a CWR-2 membrane protein was implicated to be important for allorecognition of *N. crassa*.^63^Figure S19 shows a model of a hypothetical protein–protein interaction between *Mt*LPMO9B and *Mt*AA16A. It should be emphasized that this model, though having a high score, only represents an illustrative model and other factors such as glycosylation location, CBM, and linker were not taken into account. In addition, it cannot be excluded that also other or additional pathways might be valid, such as electron transfer between the AA16 and LPMO active sites.

### 3.8 Functions of AA16 Oxidoreductases in Nature

As listed in the CAZy database, three putative AA16 proteins have been identified in the genome of *M. thermophila*, and zero candidates have been found in the genome of *N. crassa*.^6^ This observation may hint at a natural, evolution-driven difference and might relate to our results showing the interplay between the AA16s and *Mt*LPMO9s, being absent for *Nc*LPMO9s. This idea is strengthened by results from a recent study, in which Grieco et al. reported that, in the *M. thermophila* secretome, one AA9 LPMO (MYCTH_89312; *Mt*LPMO9B) was detected together with another AA16 member (MYCTH_2311254) and one AA3 CDH (MYCTH_81925), when grown on partially delignified sugarcane bagasse.^36^ CDH is a well-known electron-donating enzyme for AA9 LPMOs,^13,18,64,65^ and we here suggest that the AA16s serve as H_2_O_2_ producers, possibly even interacting with other LPMOs. In addition to the AA16s, other H_2_O_2_-producing enzymes are expected to also drive LPMO reactions, such as AA7 oligosaccharide oxidases,^66−69^ and such AA7s have also been found to be coexpressed with LPMOs.^70,71^

In this study, we were not able to find any carbohydrate substrates for oxidative cleavage by AA16s, though a very intensive substrate screening for AA16s was performed. It still cannot be excluded that AA16s are indeed LPMOs, but the biological substrates remain unknown. So far, only three AA16 members have been studied, which may not represent the complete picture of this family. Looking at the phylogenetic trees (Figures S20 and S21), AA16s show high sequence variability. Filiatrault-Chastel and co-workers reported that many AA16s have a *C*-terminal extension, CBM1, or glycosylphosphatidylinositol (GPI) anchors in addition to the catalytic domains.^11^ It is still unclear how these additional domains contribute to the AA16 functions in nature.

AA16 sequences were also found in the pathogenetic oomycetes *Phytophthora* and *Pythium* species.^11^ More recently, AA16s were shown to be the only LPMO family members that coexpressed with the newly discovered AA17 pectin-active LPMOs during the infection of potato leaves by *Phytophthora infestans*.^7^ Though the expression level was lower compared to AA17s, it still indicates that AA16s might play other roles in nature.

## 4 Conclusions

Our study has obtained insights into the catalytic and structural properties of *Mt*AA16A and *An*AA16A, members of a new family of CAZy enzymes. Although the crystallographic structure of *Mt*AA16A showed a copper-containing His-brace typical for LPMOs, the adjacent substrate-binding surface differed. In addition, both *Mt*AA16A and *An*AA16A did not oxidatively cleave any of the investigated carbohydrates. We showed that both *Mt*AA16A and *An*AA16A produced (low levels) H_2_O_2_ and stimulated the cellulolytic peroxygenase reaction of *Mt*LPMO9s. No such stimulation was observed with *Nc*LPMO9s, while both *Mt*LPMO9s and *Nc*LPMO9s were boosted by externally added H_2_O_2_. We showed that the strong AA16 boosting effect on *Mt*LPMO9B cannot be achieved using a similar H_2_O_2_-producing activity of *An*GOX. We propose that, within a hypothetical protein–protein complex, the formed H_2_O_2_ might easily reach the catalytic site of *Mt*LPMO9s, where it serves as a preferred cosubstrate to drive the peroxygenase reaction. Lastly, we discussed the possible functions of AA16s in nature, which deserve further investigation.

## Supporting Information Available

Deglycosylation of *Mt*AA16A by PNGase F; incubation of *Mt*AA16A with various carbohydrates; determination of peroxidase activity of *Mt*AA16A and *Mt*LPMO9s by the 2,6-DMP assay; binding of *Mt*AA16A to regenerated amorphous cellulose; thermal shift analysis of *Mt*AA16A interaction with cello-oligosaccharides and syringol; computational modeling of *Mt*LPMO9B–*Mt*AA16A interaction; phylogenetic analysis of the AA16 family; SDS-PAGE of *Mt*AA16A and *An*AA16A (Figure S1); multiple sequence alignment and sequence identity matrices of *Aa*AA16, *Mt*AA16A, and *An*AA16A (Figure S2); LC-MS analysis of the *N*-terminal peptide of *Mt*AA16A (Figure S3); 2,6-DMP activity of *Mt*LPMO9s and *Mt*AA16A (Figure S4); comparison of the *Mt*AA16A structure with the three highest DALI scoring structures (Figure S5); comparison of the *Mt*AA16A copper binding site with the three highest DALI scoring structures (Figure S6); percentage of unbound *Mt*AA16A protein after incubation with RAC (Figure S7); thermal denaturation of *Mt*AA16A monitored by nDSF in the presence of potential ligands (Figure S8); HPAEC chromatograms of control reactions in the presence of Pyg (Figure S9); HPAEC chromatograms of *Mt*LPMO9B-RAC digestion with or without AA16s at 2, 4, and 6 h (Figures S10); HPAEC chromatograms of RAC samples incubated with various AA9 LPMOs in the presence of 1 mM Asc after 6 h (Figure S11); calibration curves of H_2_O_2_ levels determined by the Amplex Red/HRP assay in the absence and presence of different Asc concentrations (Figure S12); HPAEC elution patterns of RAC samples incubated with only *Mt*LPMO9B, *Mt*LPMO9B with the addition of 50 μM H_2_O_2_ (in total 6 times), and *Mt*LPMO9B + *An*AA16A and *Mt*LPMO9B + *Mt*AA16A in the presence of Asc at 6 h (Figure S13); HPAEC chromatograms of *Mt*LPMO9B-RAC digestion in the presence of Asc and different concentrations of H_2_O_2_ (Figure S14); HPAEC chromatograms of control reactions in the presence of Asc (Figure S15); HPAEC chromatograms of *Nc*LPMO9M-RAC digestion in the presence of Asc and different concentrations of H_2_O_2_ (Figure S16); comparison of HPAEC chromatograms of *Nc*LPMO9M-RAC digests in the presence of Asc and addition of 10 μM H_2_O_2_ per time (6 times in total) or *Mt*AA16A or *An*AA16A (Figure S17); activity (mU) calibration curve of different concentrations of glucose oxidase from *Aspergillus niger* (*An*GOX) (Figure S18); plausible interaction surface on *Mt*LPMO9B and *Mt*AA16A (Figure S19); unrooted phylogenetic tree of full-length amino acid sequences of AA16 members (Figure S20); unrooted phylogenetic tree of catalytic domain amino acid sequences of AA16 members (Figure S21); crystallographic statistics (Table S1); carbohydrates tested for screening *Mt*AA16A activity under different conditions (Table S2); geometry at the *Mt*AA16A copper binding site (Table S3); H_2_O_2_-producing activity of *Mt*AA16A, *Mt*LPMO9s, and *Nc*LPMO9s in the presence of 50 μM Asc (Table S4); and references (PDF)
