# Supplementary material for: AA16 Oxidoreductases Boost Cellulose-Active AA9 Lytic Polysaccharide Monooxygenases from Myceliophthora thermophila
Source: ACS Catal. 2023 Mar 21;13(7):4454–67. doi: 10.1021/acscatal.3c00874 (PMC10088020; doi:10.1021/acscatal.3c00874)
Supplement: Supplementary file 1 — cs3c00874_si_001.pdf [file cs3c00874_si_001.pdf]

Supporting Information

AA16 Oxidoreductases Boost Cellulose Active AA9 Lytic Polysaccharide Monooxygenases from *Myceliophthora thermophila*

Peicheng Sun[1], Zhiyu Huang[2], Sanchari Banerjee[2], Marco A.S. Kadowaki[3], Romy J. Veersma[1], Silvia Magri[3], Roelant Hilgers[1], Sebastian J. Muderspach[2], Christophe V.F.P. Laurent[4,5], Roland Ludwig[4], David Cannella[3], Leila Lo Leggio[2], Willem J.H. van Berkel[1] and Mirjam A. Kabel[1,*]

[1]Laboratory of Food Chemistry, Wageningen University & Research, Bornse Weilanden 9, 6708 WG, Wageningen, The Netherlands

[2]Department of Chemistry, University of Copenhagen, Universitetsparken 5, 2100, Copenhagen, Denmark

[3]PhotoBioCatalysis Unit (CPBL) and Biomass Transformation Lab (BTL), École Interfacultaire de Bioingénieurs (EIB), Université Libre de Bruxelles, Avenue F.D. Roosevelt 50, 1050, Bruxelles, Belgium

[4]Biocatalysis and Biosensing Laboratory, Department of Food Science and Technology, University of Natural Resources and Life Sciences (BOKU), Muthgasse 18, 1190, Vienna, Austria

[5]Institute of Molecular Modeling and Simulation, Department of Material Sciences and Process Engineering, University of Natural Resources and Life Sciences (BOKU), Muthgasse 18, 1190, Vienna, Austria

*Corresponding author (Telephone: +31 (0)317 48 32 09, email: mirjam.kabel@wur.nl)

Number of pages: 33

Number of figures: 21

Number of tables: 4

Supporting Information contents:

Deglycosylation of *Mt*AA16A by PNGase F

Incubation of *Mt*AA16A with various carbohydrates

Determination of peroxidase activity of *Mt*AA16A and *Mt*LPMO9s by 2,6-DMP assay

Binding of *Mt*AA16A to regenerated amorphous cellulose

Thermal shift analysis of *Mt*AA16A interaction with cello-oligosaccharides and syringol

Computational modeling of *Mt*LPMO9B-*Mt*AA16A interaction

Phylogenetic analysis of AA16 family

Figure S1. SDS-PAGE of *Mt*AA16A and *An*AA16A

Figure S2. Multiple sequence alignment and sequence identity matrices of *Aa*AA16, *Mt*AA16A and *An*AA16A

Figure S3. LC-MS analysis of *N*-terminal peptide of *Mt*AA16A

Figure S4. 2,6-DMP activity of *Mt*LPMO9s and *Mt*AA16A

Figure S5. Comparison of the *Mt*AA16A structure with the three highest DALI scoring structures

Figure S6. Comparison of the *Mt*AA16A copper binding site with the three highest DALI scoring structures

Figure S7. Percentage of unbound *Mt*AA16A protein after incubation with RAC

Figure S8. Thermal denaturation of *Mt*AA16A monitored by nDSF in the presence of potential ligands

Figure S9. HPAEC chromatograms of control reactions in the presence of Pyg

Figure S10. HPAEC chromatograms of *Mt*LPMO9B-RAC digestion with or without AA16s at 2, 4 and 6 h

Figure S11. HPAEC chromatograms of RAC samples incubated with various AA9 LPMOs in the presence of 1 mM Asc after 6 h

Figure S12. Calibration curves of H_2O_2 levels determined by Amplex Red/HRP assay in the absence and presence of different Asc concentrations.

Figure S13. HPAEC elution patterns of RAC samples incubated with only *Mt*LPMO9B, *Mt*LPMO9B with the addition of 50 μM H_2O_2 (in total 6 times), *Mt*LPMO9B+*An*AA16A and *Mt*LPMO9B+*Mt*AA16A in the presence of Asc at 6 h

Figure S14. HPAEC chromatograms of *Mt*LPMO9B-RAC digestion in the presence of Asc and different concentration of H_2O_2

Figure S15. HPAEC chromatograms of control reactions in the presence of Asc

Figure S16. HPAEC chromatograms of *Nc*LPMO9M-RAC digestion in the presence of Asc and different concentration of H_2O_2

Figure S17. Comparison of HPAEC chromatograms of *Nc*LPMO9M-RAC digests in the presence of Asc and addition of 10 μM H_2O_2 per time (6 times in total) or *Mt*AA16A or *An*AA16A

Figure S18. Activity (mU) calibration curve of different concentrations of glucose oxidase from *Aspergillus niger* (*An*GOX)

Figure S19. A plausible interaction surface on *Mt*LPMO9B and *Mt*AA16A

Figure S20. Unrooted phylogenetic tree of full-length amino acid sequences of AA16 members

Figure S21. Unrooted phylogenetic tree of catalytic domain amino acid sequences of AA16 members

Table S1. Crystallographic statistics

Table S2. Carbohydrates tested for screening *Mt*AA16A activity under different conditions

Table S3. Geometry at the *Mt*AA16A copper binding site

Table S4. H_2O_2 producing activity of *Mt*AA16A, *Mt*LPMO9s and *Nc*LPMO9s in the presence of 50 μM Asc.

References

Deglycosylation of *Mt*AA16A by PNGase F

To remove *N*-glycosides, 40 µg of *Mt*AA16A was incubated with PNGase F (*N*-Glycosidase F, Madison, Wisconsin, USA) to remove *N*-glycosylation. The deglycosylation procedure was performed using non-denaturing conditions based on the protocol provided by the manufacturer. 4 µL PNGase F was added to 36 µL 50 mM ammonium bicarbonate (pH 7.8) containing 40 µg of *Mt*AA16A. The sample was incubated at 37 °C for 16 h under shaking of 600 rpm (Eppendorf ThermoMixer C, Eppendorf, Hamburg, Germany).

Incubation of *Mt*AA16A with various oligo and polysaccharides

Carbohydrates (2 mg/mL each, types and sources are listed in Table S2) were dissolved in 50 mM ammonium acetate buffer (pH 5.0) with the addition of reducing agent (Table S2, 1 mM final concentration). Subsequently, *Mt*AA16A was added to a concentration of 20 or 40 mg/g carbohydrate (Table S2). Control reactions were performed without the addition of reducing agent. The incubation of 500 mL was performed at 30 or 50 °C (Table S2) for 24 h in an Eppendorf ThermoMixer C at 900 rpm (in a vertical orientation). In some reactions, 100 µM H_2O_2 was added in the beginning of incubation. The incubations were stopped by immediately separating supernatants and pellets through centrifugation 22,000 ×*g*, 15 min at 4 °C in a table centrifuge. The supernatants were used for immediate further analysis or stored at -20 °C.

Determination of peroxidase activity of *Mt*AA16A and *Mt*LPMO9s by 2,6-DMP assay

A spectrophotometric assay using 2,6-dimethoxyphenol (2,6-DMP assay) was performed according to Breslmayr et al.[1] The assay was carried out in a 96-well plate with total reaction volumes of 250 µL. Each reaction contained 100 mM potassium phosphate buffer pH 6.0, 10 mM 2,6-DMP and 100 µM H_2O_2.[1] After pre-incubation at 30 °C, *Mt*LPMO9B, *Mt*LPMO9E, *Mt*LPMO9H, *Mt*LPMO9I or *Mt*AA16A were added to give a final concentration of 0.405 mg/mL, 0.49 mg/mL, 0.401 mg/mL, 0.448 mg/mL and 0.73 mg/mL, respectively. The control reaction was performed without enzymes. After the addition of enzyme, the 96-well plate was placed in a spectrophotometer (Infinite F500, Tecan, Männedorf, Switzerland) and the absorbances were recorded at 480 nm for 600 s at 30 °C. The molar absorption coefficient of the reaction product coerulignone (53,200 M^{-1} cm^{-1}) and the path length of 0.7 cm were used to calculate the specific activities of the *Mt* enzymes. 1 unit (U) of enzyme activity is defined as the amount of enzyme that converts 2 µmol 2,6-DMP into coerulignone per min.

Binding of *Mt*AA16A to regenerated amorphous cellulose

Binding experiments were performed according to Sun et al.[2] 5 µM *Mt*AA16A were added to 2 mg/mL regenerated amorphous cellulose (RAC) in 50 mM ammonium acetate buffer pH 5.0. The protein-cellulose mixture (1.2 mL) was incubated at 30 °C in an Eppendorf ThermoMixer Comfort (Eppendorf, Hamburg, Germany) at 800 rpm (in an almost vertical orientation). At 30, 60, 90, 120, 150 and 180 min, 200 µL of sample was taken out from the tubes. Subsequently, supernatant with free protein was separated and collected from residual cellulose with bound protein through centrifugation 22,000 ×*g*, 2 min at 4 °C in a table centrifuge. The amount of bound protein was calculated based on the following equation (eq.1):

Eq. 1 $Bound\ protein\ (\mu M) = Total\ protein\ (\mu M) - Total\ protein\ (\mu M) \times \frac{Abs\ free\ (AU\ 280)}{Abs\ total\ (AU\ 280)}$

where *Total protein* = 5 µM; *Abs free* = absorbance of free protein in the supernatant at 280 nm; *Abs total* = absorbance of total protein solution without substrate at 280 nm.

The absorbance of free protein and total protein was measured spectrophotometrically at 280 nm in a UV-compatible 96-well plate. Incubations were performed in triplicate.

Thermal shift analysis of *Mt*AA16A interaction with cello-oligosaccharides and syringol

A Tycho-NT6 device (NanoTemper Technologies GmbH, München, Germany) was used to probe ligand binding by thermal shift assays. 20 mg/ml *Mt*AA16A in the sample buffer was first pre-incubated with equimolar Cu (II) acetate for 1 h at 4°C. Tested ligands were cellobiose (Cell2), cellotriose (Cell3), cellotetraose (Cell4), cellopentaose (Cell5) and cellohexaose (Cell6) (Megazyme) and syringol (Sigma-Aldrich) solubilized in 20 mM sodium acetate buffer, pH 5.5. *Mt*AA16A (final concentration 2 mg/mL) was incubated with ligand for 5 min in 20 mM sodium acetate (pH 5.5) buffer before the measurement. For the measurement, 10µL mixture was loaded onto a capillary and tested in the device. The temperature was increased from 35 to 95 °C in 3 min to monitor the ratio of intrinsic fluorescence of aromatic residues at 350 and 330 nm. The inflection point (°C) was determined using the instrument software.

Computational modeling of *Mt*LPMO9B-*Mt*AA16A interaction

The *Mt*LPMO9B structure was modeled by AlphaFold 2 based on Uniprot ID G2QCJ3 after removal of the predicted signal peptide. Surface patches likely involved in *Mt*LPMO9B-*Mt*AA16A protein-protein interaction were identified using the InterProSurf server (http://curie.utmb.edu/prosurf.html).[3] Aromatic patches were identified in PyMOL. HADDOCK v 2.4[4] was used for modeling of the proposed protein complex. Figures were rendered in PyMOL (v2.0.1 2018, Schrödinger, Inc., NY, USA).

Phylogenetic analysis of AA16 family

The amino acid sequences of 82 AA16 candidates were downloaded from the National Center for Biotechnology Information (NCBI) database (https://www.ncbi.nlm.nih.gov). Four amino acid sequences were found to be incomplete as *N*-terminal histidine was missed. After removing signal peptides determined by SignalP 6.0,[5] the remaining 78 sequences were aligned using Multiple Alignment using Fast Fourier Transform (MAFFT).[6] The alignment was subjected to phylogenetic analysis using unrooted Neighbor Joining (NJ) algorithm with 500 bootstraps in MEGA7 software (MEGA Limited, Auckland, New Zealand).[7] The resulting phylogenetic tree was referred as full-length. Based on the MAFFT alignment, the amino acid sequences were fine-tuned by cutting-out the C-terminal extension, linker, CBM or other non-catalytic domains. The amino acid sequences of the remaining catalytic domain were realigned using MAFFT, and the resulting alignment was used to build a catalytic-domain phylogenetic tree as described above.

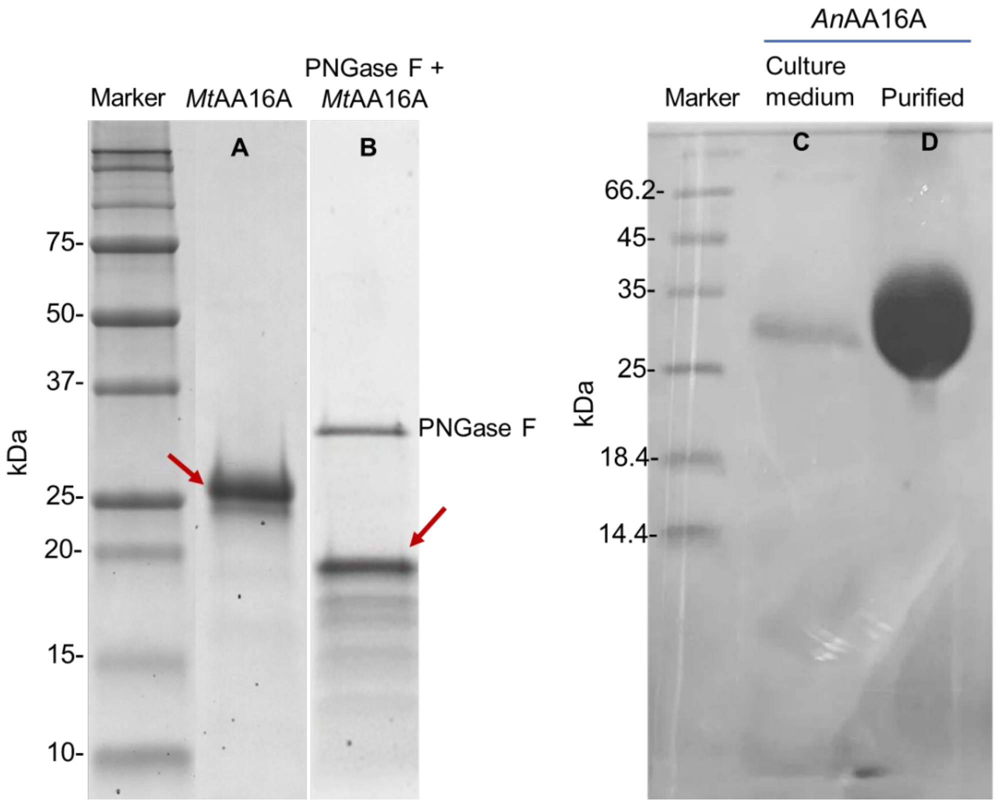

Figure S1. SDS-PAGE of (A) purified *Mt*AA16A, (B) deglycosylated *Mt*AA16A, (C) culture medium of *An*AA16A and (D) purified *An*AA16A. *Mt*AA16A bands are marked with red arrow.

A

```
          1         11        21        31        41        51
          |         |         |         |         |         |
AaAA16    HGFVTSPQPRMPGSAMEKACGQQVYNNQEADNYGNIQGELQIASGQSDYDAEACDIWLCK
AnAA16A   HGYISSPQPRMPGSAMAAACGQQVYNNQAADRAGNVQGELQVAASQSDYDADACHIWLCK
MtAA16A   HAVVTVPTPRGAGPYYTQRCGETYAVYMEKDKAGPIENGVAKAG-----SELGCNPFLCR
Cons      *. :: * **  *      **:        *. * ::. :  *.     .  .*. :**:

          61        71        81        91        101       111
          |         |         |         |         |         |
AaAA16    GYKYADNTANVQSYKPGEVIDFTVDIRA-PHTGTANVSVVDTATNTMLSQPLIYWSVYAS
AnAA16A   GYQFDDNTDNVQSYTAGETVDFVIDIVA-PHSGVANVSVVDTASNSVIGSALKSWDVYAS
MtAA16A   GYQYEDNEA--VEYEPGQVIDFHVDLIAGHHPGYANVSIVDLEANKIIGDPLRSWDDYPN
Cons      **:: **     .*  *:.:** :*: *  * * ****:**  :*.::.. *  *. * .

          121       131       141       151       161       171
          |         |         |         |         |         |
AaAA16    TATGVTANETSFSVTMPTDLGDKCNEAGACVLQWWWDARSIDQTYESCVDFTLSG
AnAA16A   TETGVTEDETNFSITIPDNLGSQCSEAGACVLQWYWYAESIDQTYESCVDFTVGG
MtAA16A   ATATTPRSDIDFNVTIPNTLGTACSTGGKCAIQWYWYASGNKQSYESCVDFYVKA
Cons      : : .  .: .*.:*:*  **  *. .* *.:**:* * . .*:******* : .
```

B

Full sequence

*Aa*AA16	100%		
*An*AA16A	60.8%	100%	
*Mt*AA16A	40.1%	39.5%	100%
	*Aa*AA16	*An*AA16A	*Mt*AA16A

Catalytic domain

*Aa*AA16	100%		
*An*AA16A	69.5%	100%	
*Mt*AA16A	40.1%	39.5%	100%
	*Aa*AA16	*An*AA16A	*Mt*AA16A

Figure S2. (A) Multiple sequence alignment of *Aa*AA16 (without C-terminal extension), *An*AA16A (without C-terminal extension) and *Mt*AA16A using Clustal Omega tool from the EMBL-EBI (www.ebi.ac.uk) with the default parameters. Histidine residues involved in copper ion coordination are marked in yellow. The glycosylation site of *Mt*AA16A is marked in magenta. (B) Sequence identity matrices of *Aa*AA16, *An*AA16A and *Mt*AA16A based on the full amino acid sequence (left) or the amino acid sequence of catalytic domain (right).

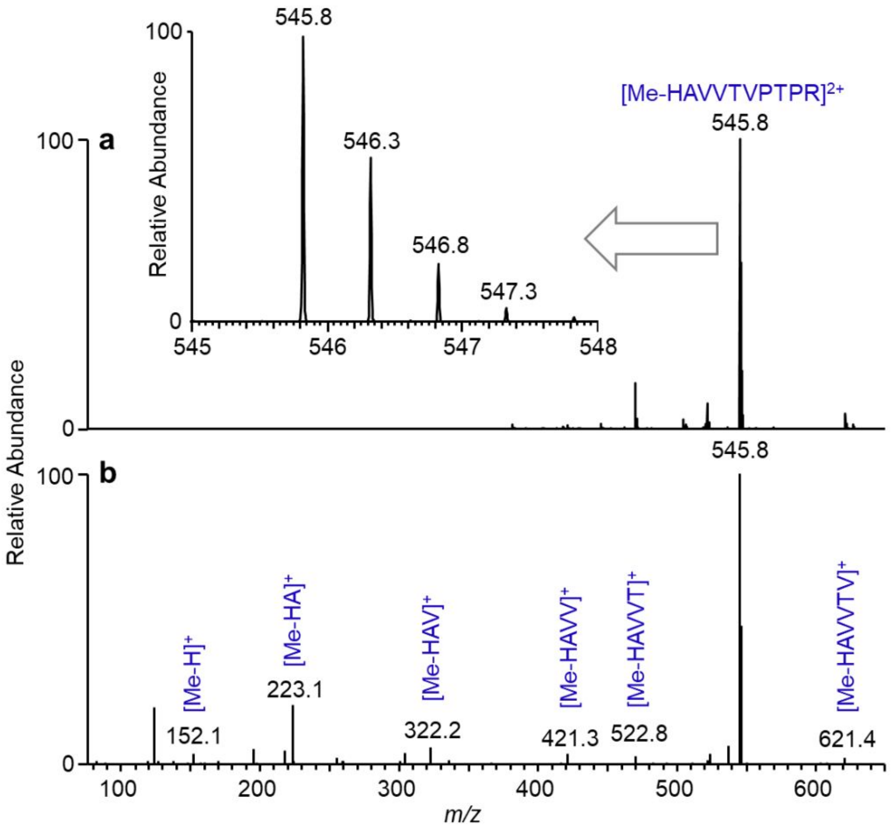

Figure S3. LC-MS analysis of the *N*-terminal peptide of *Mt*AA16A using positive mode. (A) Full MS showed that the *N*-terminal peptide was double charged. (B) MS/MS^2 fragmentation patterns confirmed that the *N*-terminal peptide of *Mt*AA16A was methylated.

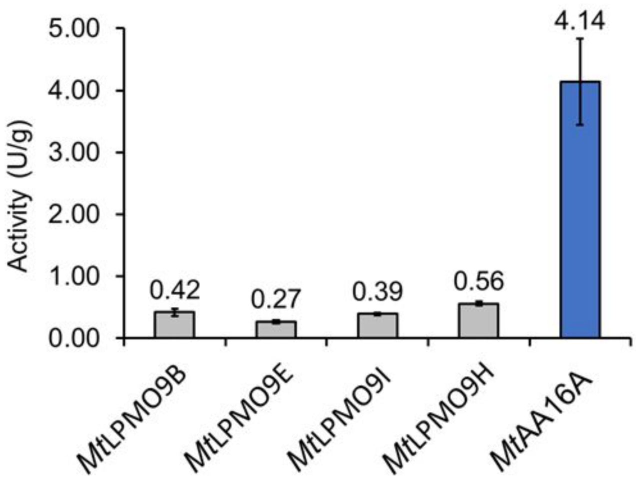

Figure S4. Specific activities of *Mt*LPMO9s and *Mt*AA16A determined by using the 2,6-DMP assay. For *Mt*AA16A, a copper-saturated fraction was used. Specific activities of *Mt*LPMO9s were comparable before and after copper-saturation. 1 unit (U) of enzyme activity is defined as the amount of enzyme that converts 2 µmol 2,6-DMP into coerulignone per min.

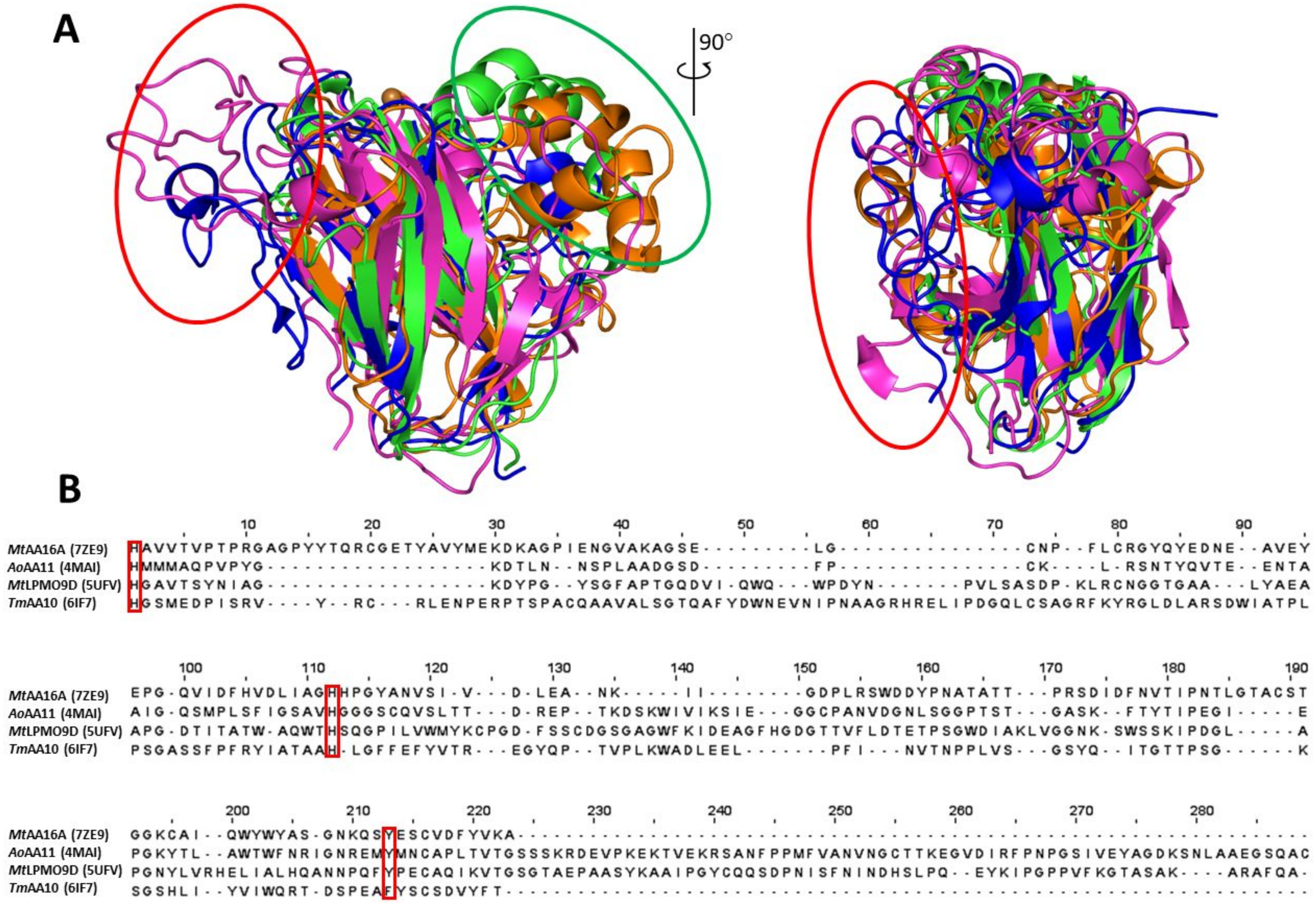
A
90°
B
MtAA16A (7ZE9) HAVVTVPTPRGAGPYYTQRCGETYAVYMEKDKAGPIENGVAKAGSE---------LG---------------CNP--FLCRGYQYEDNE--AVEY
AoAA11 (4MAI) HMMMAQPVPYG------------------KDTLN--NSPLAADGSD---------FP---------------CK----L-RSNTYQVTE--ENTA
MtLPMO9D (5UFV) HGAVTSYNIAG------------------KDYPG--YSGFAPTGQDVI-QWQ---WPDYN-------PVLSASDP-KLRCNGGTGAA---LYAEA
TmAA10 (6IF7) HGSMEDPISRV----Y--RC---RLENPERPTSPACQAAVALSGTQAFYDWNEVNIPNAAGRHRELIPDGQLCSAGRFKYRGLDLARSDWIATPL
MtAA16A (7ZE9) EPG-QVIDFHVDLIAGHHPGYANVSI-V---D-LEA--NK-----II--------GDPLRSWDDYPNATATT---PRSDIDFNVTIPNTLGTACST
AoAA11 (4MAI) AIG-QSMPLSFIGSAVHGGGSCQVSLTT---D-REP--TKDSKWIVIKSIE---GGCPANVDGNLSGGPTST---GASK--FTYTIPEGI-----E
MtLPMO9D (5UFV) APG-DTITATW-AQWTHSQGPILVWMYKCPGD-FSSCDGSGAGWFKIDEAGFHGDGTTVFLDTETPSGWDIAKLVGGNK-SWSSKIPDGL-----A
TmAA10 (6IF7) PSGASSFPFRYIATAAH-LGFFEFYVTR---EGYQP--TVPLKWADLEEL-------PFI---NVTNPPLVS---GSYQ--ITGTTPSG------K
MtAA16A (7ZE9) GGKCAI--QWYWYAS-GNKQSYESCVDFYVKA--
AoAA11 (4MAI) PGKYTL--AWTWFNRIGNREMYMNCAPLTVTGSSSKRDEVPKEKTVEKRSANFPPMFVANVNGCTTKEGVDIRFPNPGSIVEYAGDKSNLAAEGSQAC
MtLPMO9D (5UFV) PGNYLVRHELIALHQANNPQFYPECAQIKVTGSGTAEPAASYKAAIPGYCQQSDPNISFNINDHSLPQ--EYKIPGPPVFKGTASAK----ARAFQA-
TmAA10 (6IF7) SGSHLI--YVIWQRT-DSPEAFYSCSDVYFT---

Figure S5. Comparison of the *Mt*AA16A structure with the three highest DALI scoring structures. The structures with highest structure similarity to *Mt*AA16A were identified using DALI.[8, 9] (A) the structural alignment of *Mt*AA16A (green, Protein Data Bank (PDB) ID 7ZE9 Chain B), *Mt*LPMO9D (magenta, PDB ID 5UFV Chain E, DALI Z score: 10.7, RMSD of 2.9 Å on Cα atoms over 122 aligned residues), a fern AA10 from *Tectaria macrodonta, Tm*AA10 (orange, PDB ID 6IF7, DALI Z score: 10.6, RMSD of 3.2 Å on Cα atoms over 132 aligned residues) and a fungal AA11 from *Aspergillus oryzae, Ao*AA11 (blue, PDB ID 4MAI, DALI Z score: 10.2, RMSD of 2.9 Å on Cα atoms over 121 aligned residues) is shown from two directions. The areas with highest variations are highlighted. β-strand 2 of the typical β-sandwich structure is almost abolished but the corresponding extended L2 loop of *Mt*AA16A contains three small α-helices which are also observed in *Tm*AA10 (green circled region). The red circled region show the extended C-terminal in 4MAI and 5UFV compared to *Mt*AA16A which is the main contributor to their larger sizes. (B) The sequence alignment between *Mt*AA16A, *Ao*AA11, *Mt*LPMO9D and *Tm*AA10 is shown. The alignment was made using Kalign v3.3.1[10] and depicted in Jalview 2.11.1.7.[11] The histidine brace and active site tyrosine/phenylalanine are highlighted in red.

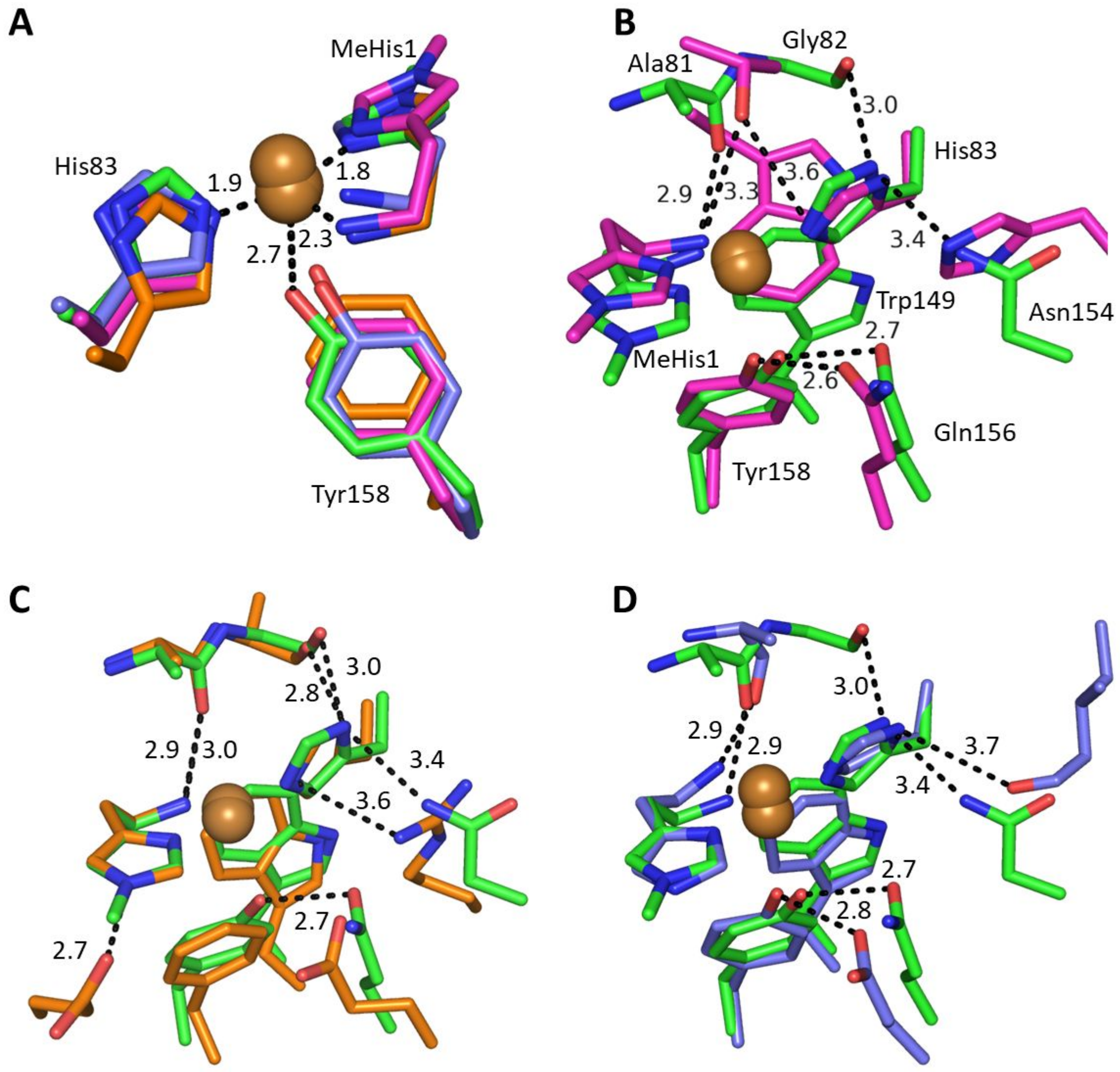

Figure S6. Comparison of the *Mt*AA16A copper binding site with those the three highest DALI scoring structures. (A) The structural alignment of the primary coordination sphere in *Mt*AA16A (green, PDB ID 7ZE9 Chain B), *Mt*LPMO9D (magenta, PDB ID 5UFV Chain E), *Tm*AA10 (orange, PDB ID 6IF7) and *Ao*AA11 (blue, PDB ID 4MAI) is shown. Structural alignment of the primary and secondary coordination sphere in *Mt*AA16A with structurally corresponding residues in *Mt*LPMO9D (B), *Tm*AA10 (C) and *Ao*A11 (D). Residues are shown as sticks, with residue numbers for *Mt*AA16A. Possible hydrogen bonds are shown as dashed lines. The aromatic platform of Trp149 is roughly 5 Å from the aromatic platform of Tyr158 and π-π interactions can be made. *Mt*AA16A shares the Gln in the second coordination sphere with AA9 and the Trp interaction with the active site Tyr with AA10 and AA11 family members.

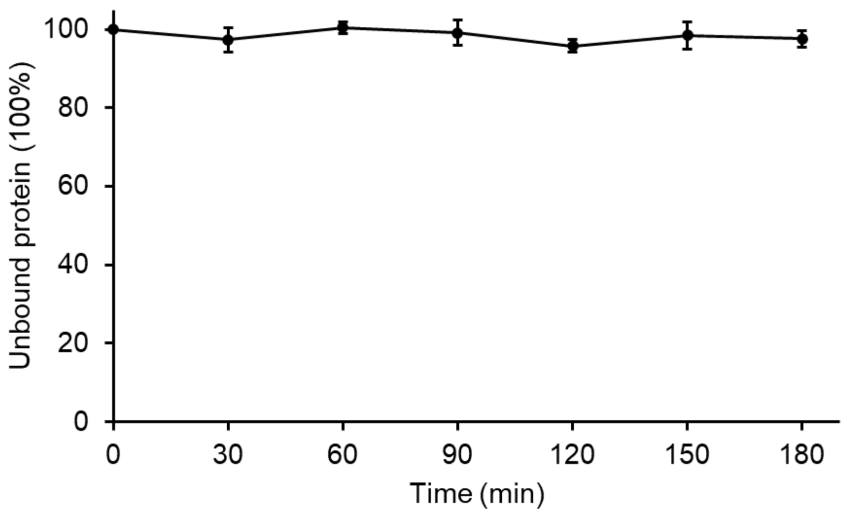

Figure S7. Percentage of unbound *Mt*AA16A protein after incubation with RAC determined by measuring absorbance at 280 nm. The amount of *Mt*AA16A in the solution before incubation with RAC was set as 100%.

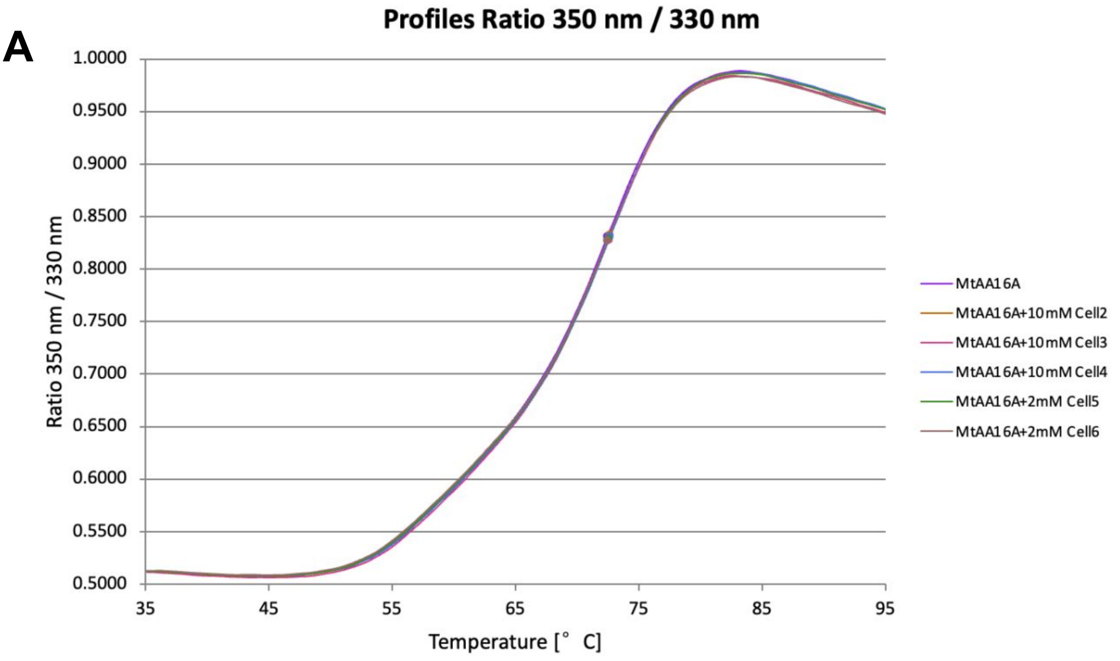

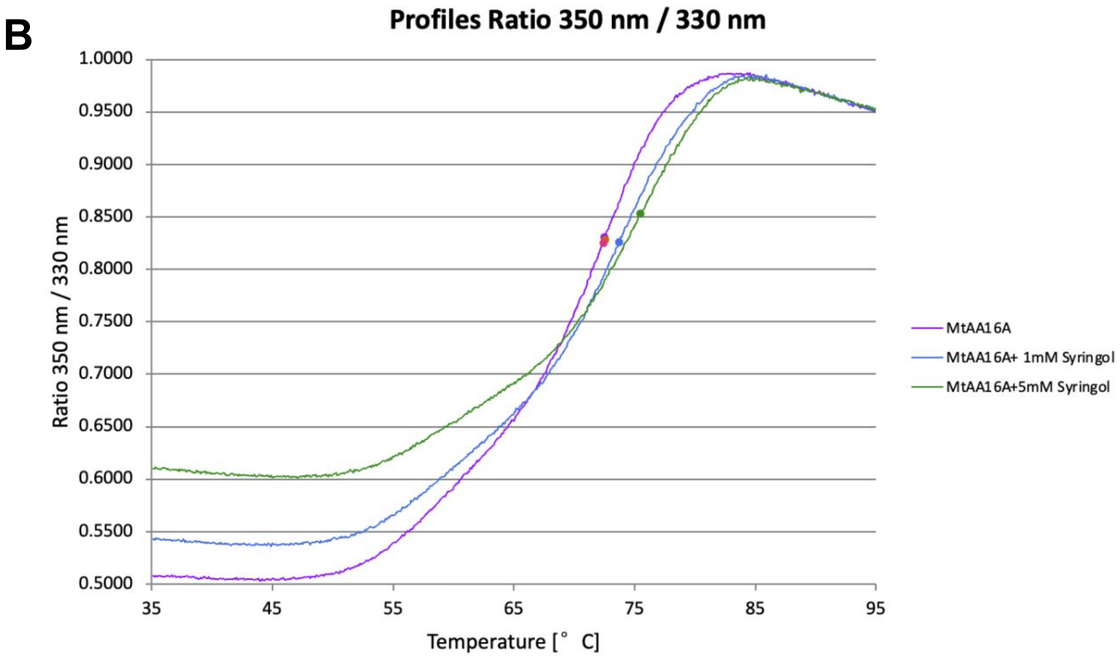

Sample	Inflection point/°C
*Mt*AA16A	72.5 ± 0.1
*Mt*AA16A + 1 mM Syringol	73.6 ± 0.1
*Mt*AA16A + 5 mM Syringol	75.3 ± 0.5

Figure S8. Thermal denaturation of *Mt*AA16A monitored by nDSF in the presence of potential ligands. (A) Cello-oligosaccharides produce no thermal shift. (B) Syringol produces a clear thermal shift.

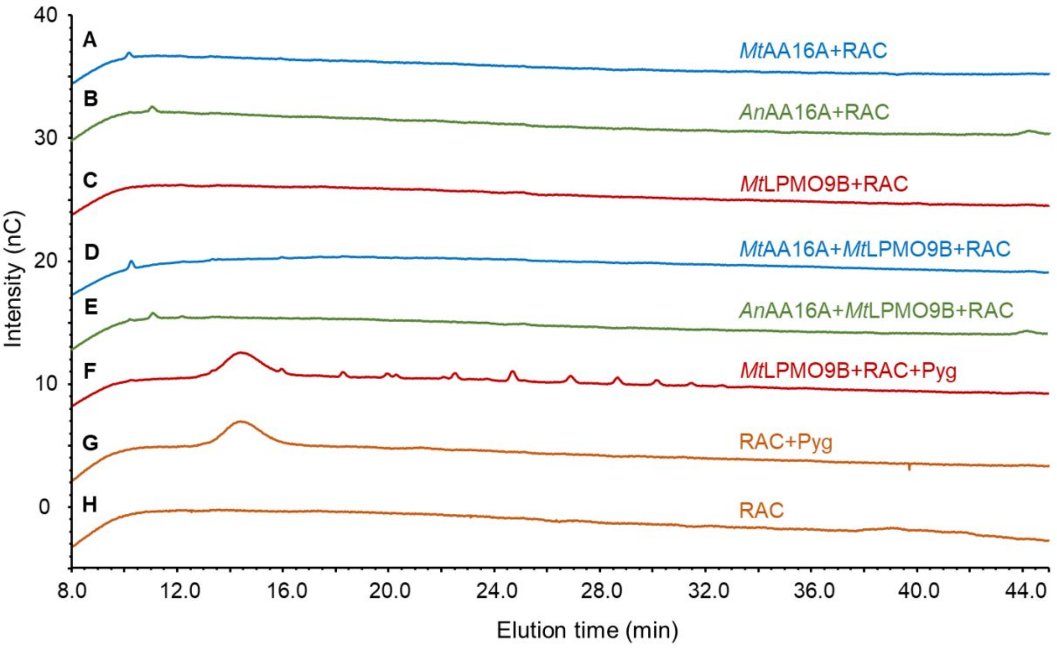

Figure S9. HPAEC chromatograms of control reactions (in the absence or presence of 1 mM Pyg). The incubation of *Mt*AA16A (A), *An*AA16A (B), *Mt*LPMO9B (C), *Mt*AA16A+*Mt*LPMO9B (D) and *An*AA16A+*Mt*LPMO9B (E) with RAC in the absence of 1 mM Pyg did not show the generation of (detectable) cello-oligosaccharides, indicating the absence of hydrolytic side-activity. (F) The incubation of *Mt*LPMO9B with RAC in the presence of Pyg showed the formation of both non-oxidized and oxidized cello-oligosaccharides. The incubation of only RAC in the presence of Pyg (G) did not cause the auto-degradation of RAC (H).

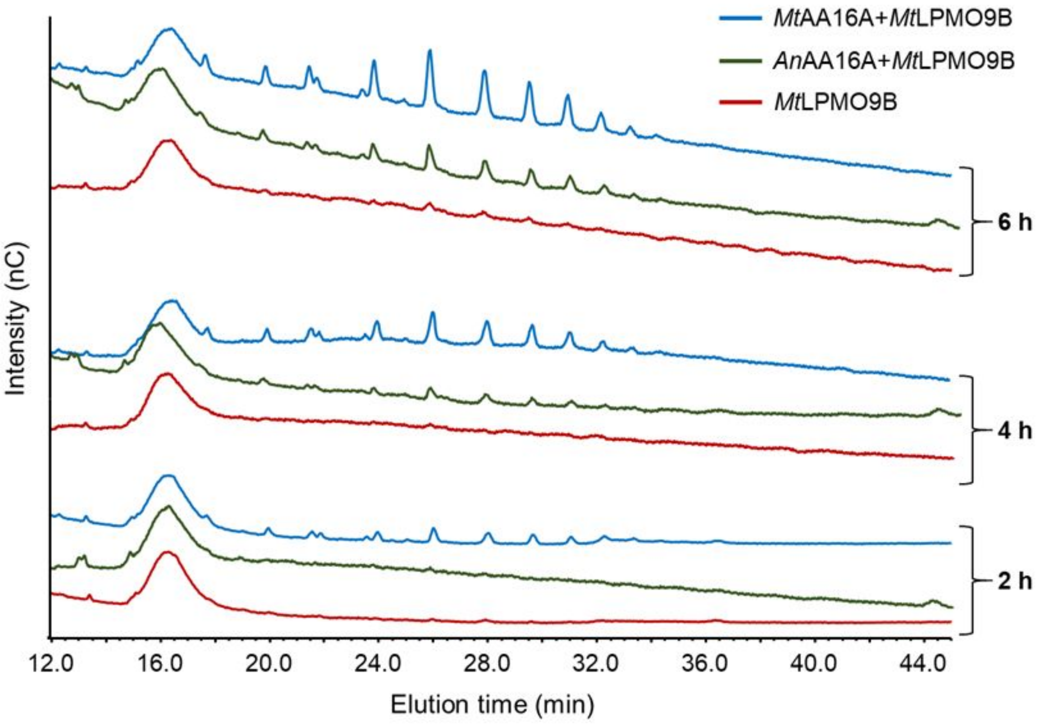

Figure S10. Oligosaccharide elution patterns determined by HPAEC. RAC samples incubated for 2, 4 and 6 h with only *Mt*LPMO9B (red line), *An*AA16A+*Mt*LPMO9B (green line) and *Mt*AA16A+*Mt*LPMO9B (blue line) in the presence of 1 mM Pyg.

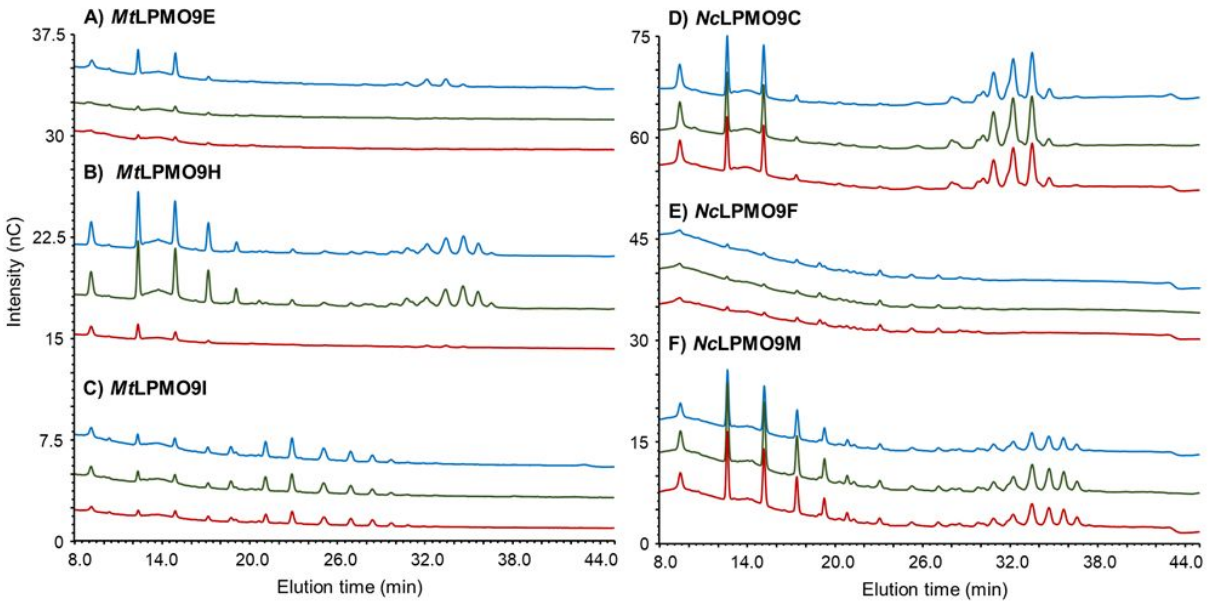

Figure S11. HPAEC chromatograms of RAC samples incubated with various AA9 LPMOs ((A) *Mt*LPMO9E; (B) *Mt*LPMO9I; (C) *Mt*LPMO9H; (D) *Nc*LPMO9C; (E) *Nc*LPMO9M and (F) *Nc*LPMO9F) in the presence of 1 mM Asc after 6 h. RAC samples incubated with only LPMO, LPMO+*An*AA16A and LPMO+*Mt*AA16A are shown in red, green and blue lines, respectively. Signal intensity of each peak and elution profiles in duplicated incubations are comparable, and only one chromatogram is shown here.

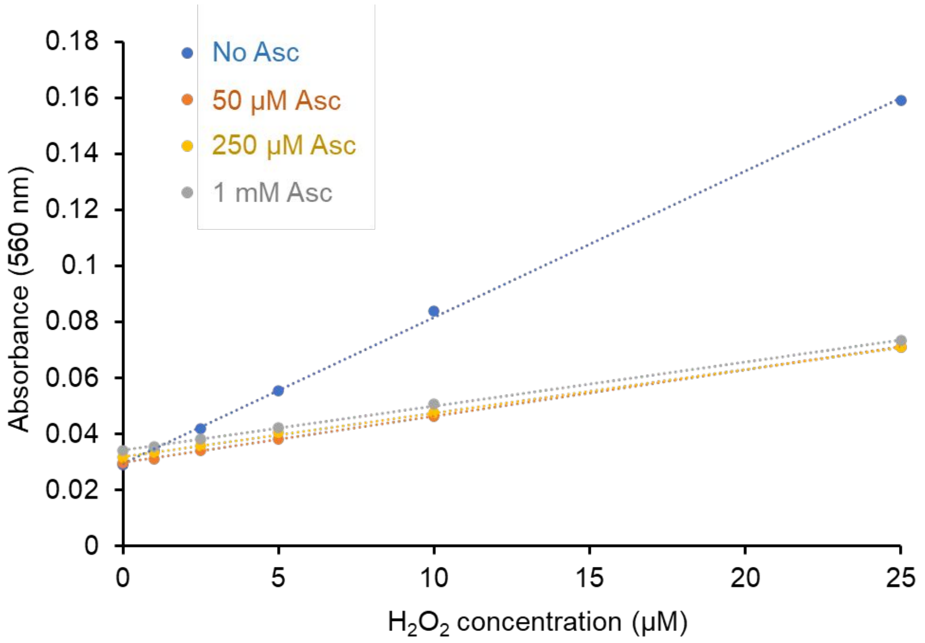

Figure S12. Calibration curves of H_2O_2 levels determined by Amplex Red/HRP assay in the absence and presence of different Asc concentrations. The absorbance (560 nm) was measured at 30 min according to the manufacturer protocol.

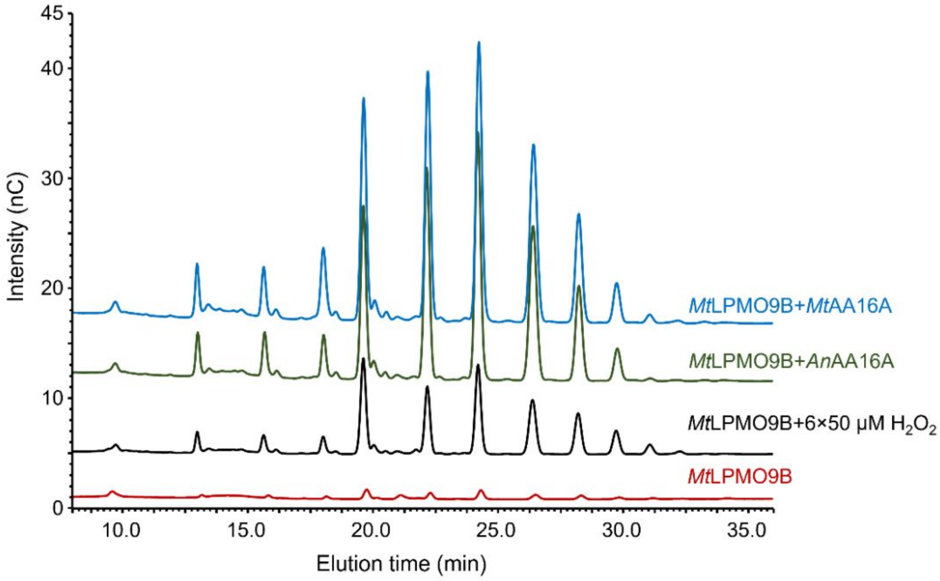

Figure S13. HPAEC elution patterns of RAC samples incubated with only *Mt*LPMO9B (red line), *Mt*LPMO9B with the addition of 50 μM H_2O_2 (50 μM each time per hour, in total 6 times) (black line), *Mt*LPMO9B+*An*AA16A (green line) and *Mt*LPMO9B+*Mt*AA16A (blue line) in the presence of 1 mM Asc at 6 h. HPAEC chromatograms of control samples are shown in Figure S9. Among all concentrations of H_2O_2, highest activity was found when adding 50 μM H_2O_2 to *Mt*LPMO9B-RAC digest at both 6 and 16 h. HPAEC chromatograms of *Mt*LPMO9B with the addition of 0, 10, 25, 100 and 200 μM H_2O_2 each time per hour (in total 6 times) in the presence of Asc are shown in Figure S14.

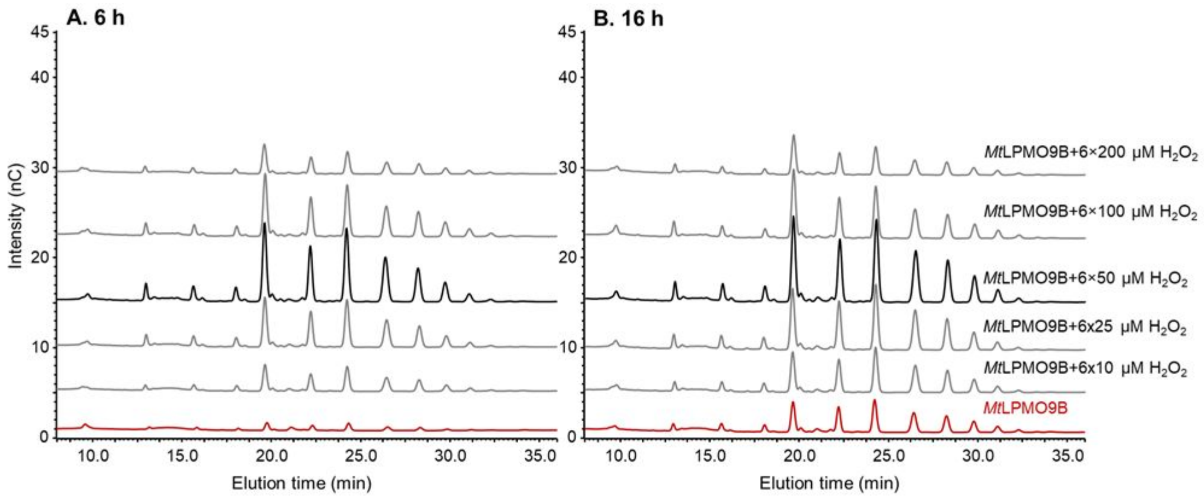

Figure S14. HPAEC chromatograms of *Mt*LPMO9B-RAC digests in the presence of 1 mM Asc and addition of different concentration of exogenous H_2O_2 (0, 10, 25, 50, 100 and 200 μM per addition per hour, 6 times in total) at 6 h (A) and 16 h (B).

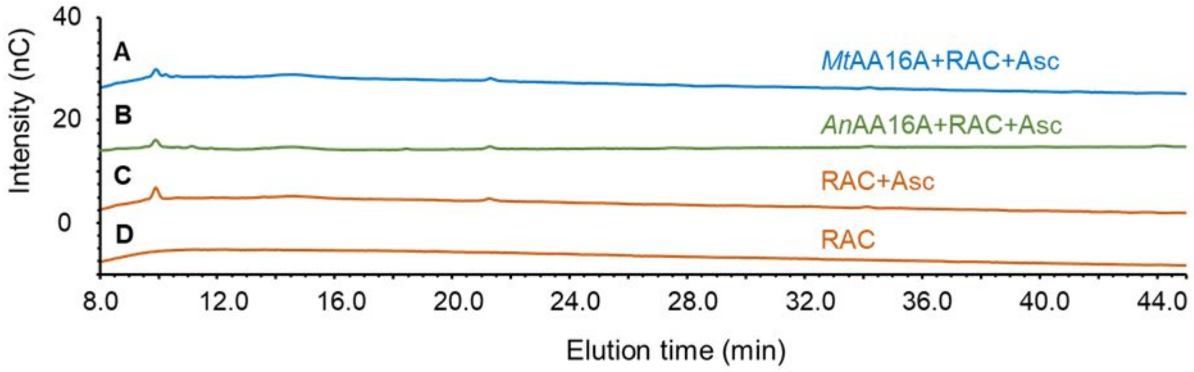

Figure S15. HPAEC chromatograms of control reactions in the presence of 1 mM Asc. The incubation of *Mt*AA16A (A) and *An*AA16 (B) with RAC in the presence of 1 mM Asc did not show the generation of (detectable) cello-oligosaccharides, indicating that these AA16s are not active towards RAC. The incubation of only RAC in the presence of 1 mM Asc (C) did not cause the auto-degradation of RAC (D).

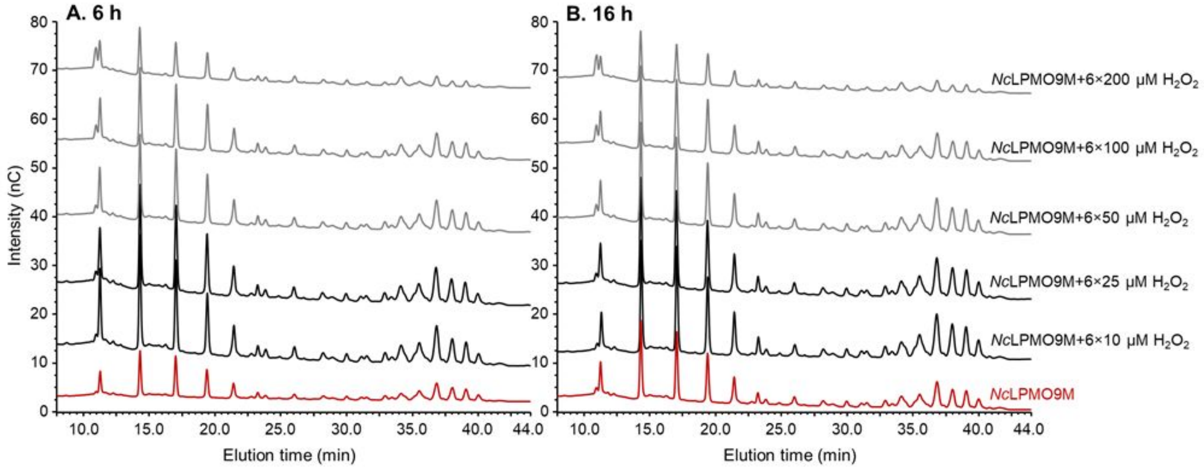

Figure S16. HPAEC chromatograms of *Nc*LPMO9M-RAC digests in the presence of 1 mM Asc and addition of different concentration of exogenous H_2O_2 (0, 10, 25, 50, 100 and 200 μM per addition per hour, 6 times in total) at 6 h (A) and 16 h (B).

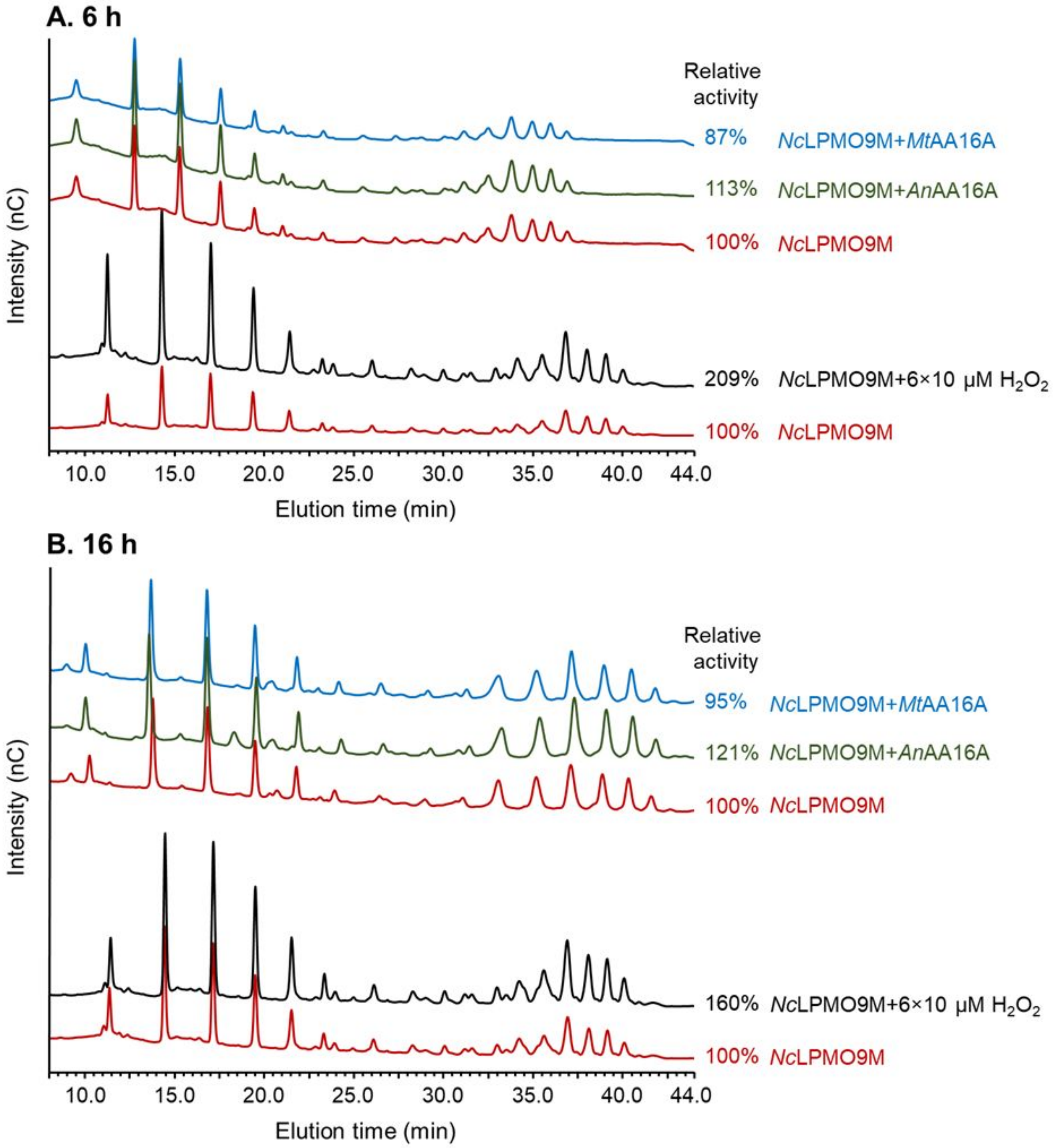

Figure S17. Comparison of HPAEC chromatograms of *Nc*LPMO9M-RAC digests in the presence of 1 mM Asc and addition of 10 μM H_2O_2 per time per hour (6 times in total) or *Mt*AA16A or *An*AA16A at (A) 6 and (B) 16 h. These chromatograms are also shown in Figure 5 and S16. Due to the analysis was performed at different systems, elution time of individual peaks is slightly different. Relative activity was calculated based on the total peak area of oxidized products. Total peak area of *Nc*LPMO9M samples at 6 and 16 h was set as 100%, respectively, and relative activities of other samples at 6 and 16 h were expressed accordingly.

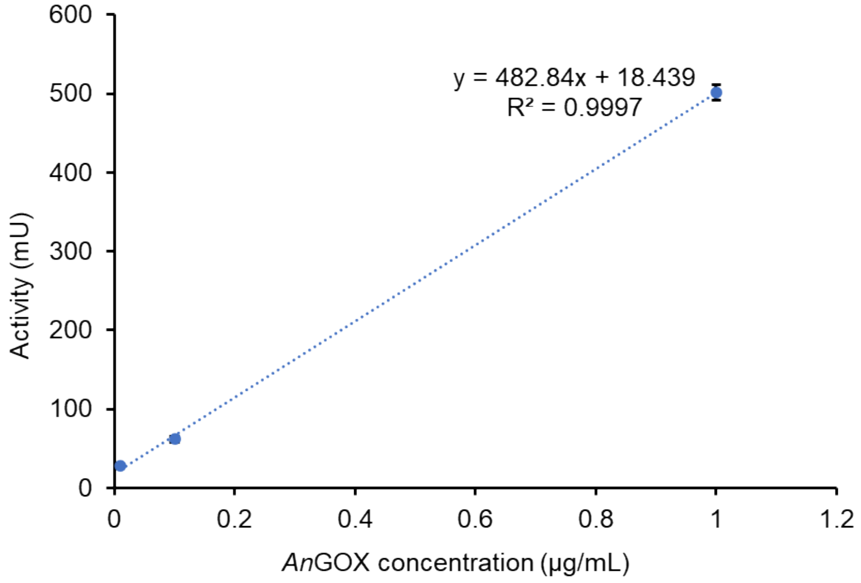

Figure S18. Activity (mU) calibration curve of different concentrations of glucose oxidase from *Aspergillus niger* (*An*GOX). The activity (mU) of each concentration was calculated based on the slope of linearly increased H_2O_2 concentration range in the reaction determined by Amplex Red/HRP assay as described in the Experimental Section. 1 U was defined as 1 µmol H_2O_2 formed per minute under the assay conditions. Based on the curve, 0.12 µg/mL *An*GOX had the activity 76.38 mU.

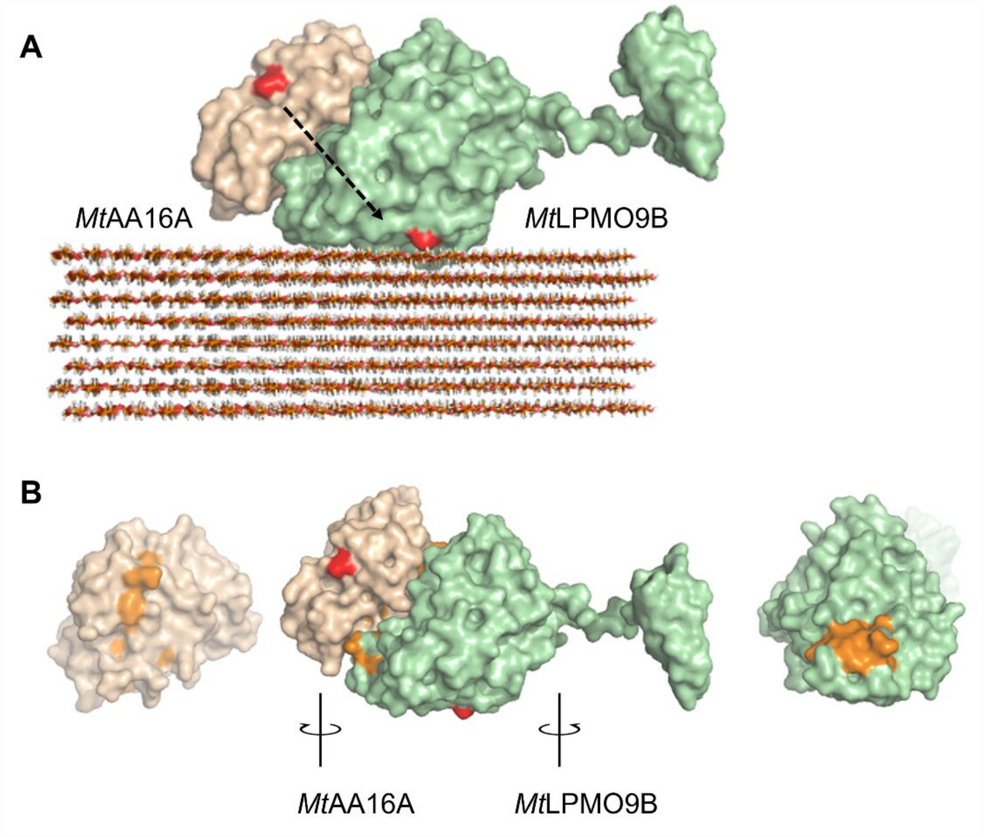

Figure S19. (A) Illustration of a possible interaction surface between *Mt*LPMO9B (pale green) and *Mt*AA16A (wheat) which allows for *Mt*LPMO9B binding on cellulose (based on the known binding mode of *Ls*AA9A on cello-oligosaccharides). His1 on *Mt*AA16A is marked red. H_2O_2 produced by *Mt*AA16A can diffuse towards the cellulose surface and active site of *Mt*LPMO9B (direction of the arrow) and thus accelerate the LPMO reaction. The illustration is based on a high scoring docking model produced in HADDOCK; (B) illustrates in orange the InterProSurf identified regions used as interacting residues in HADDOCK. The docking does not take glycosylation of either proteins into account.

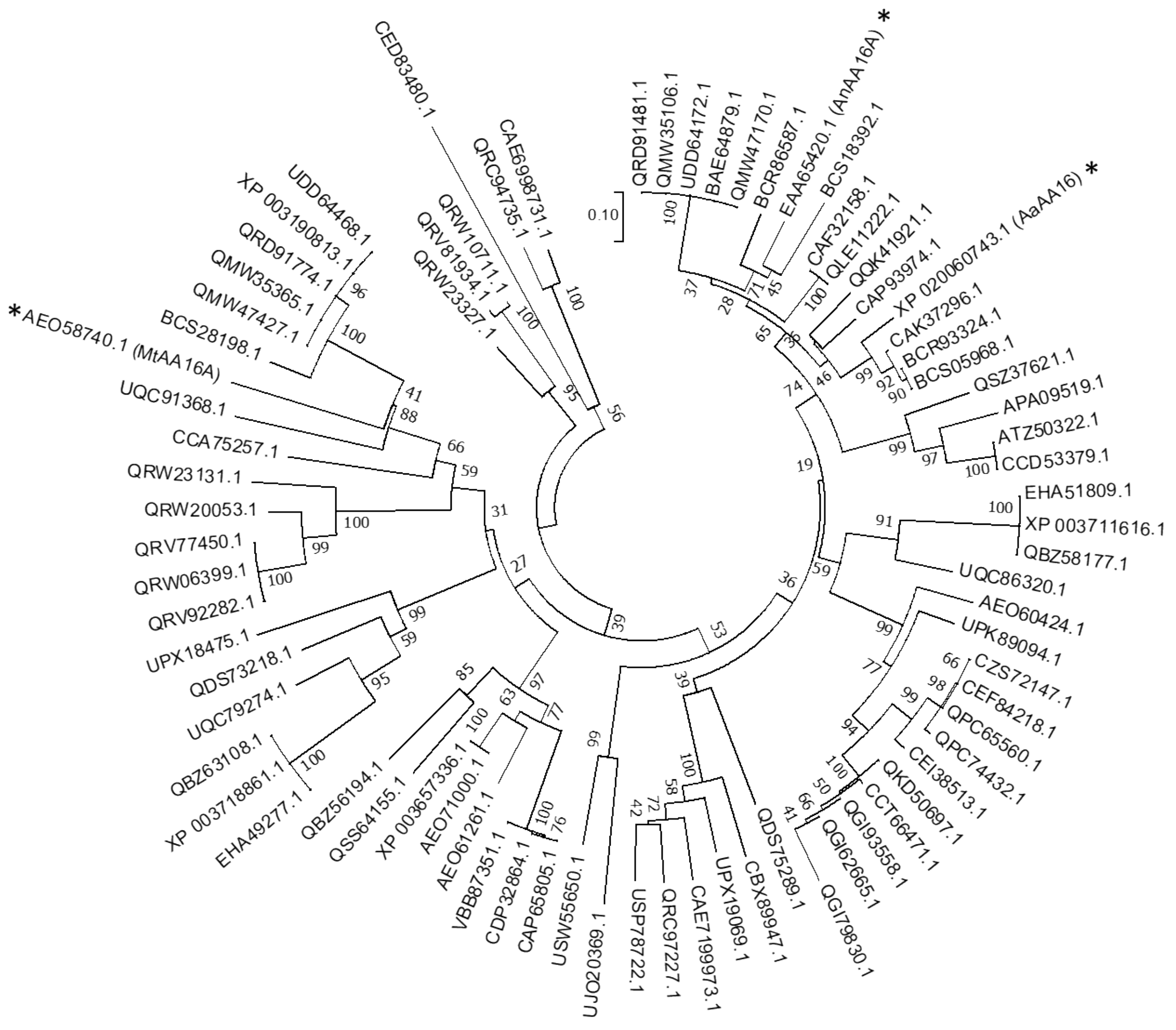

Figure S20. Unrooted phylogenetic tree of full-length amino acid sequences of AA16 members. Bootstrap values are shown at the nodes based on the Neighbor Joining algorithm. *Aa*AA16, *Mt*AA16A and *An*AA16A are marked with asterisks.

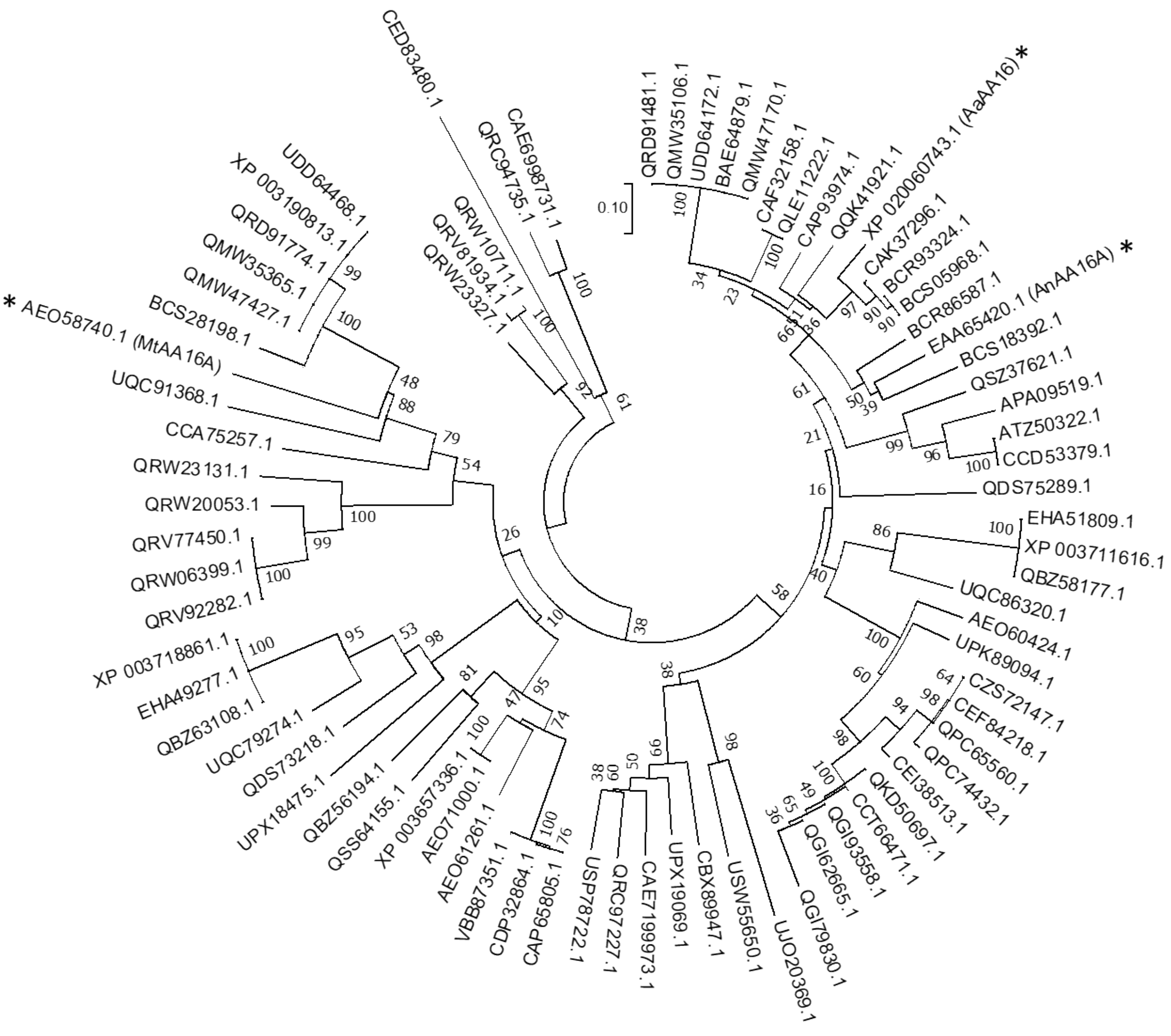

Figure S21. Unrooted phylogenetic tree of only catalytic domain amino acid sequences of AA16 members. Bootstrap values are shown at the nodes based on the Neighbor Joining algorithm. *Aa*AA16, *Mt*AA16A and *An*AA16A are marked with asterisks.

Table S1. Crystallographic statistics *The values in parentheses are for the highest resolution shell; #Ramachandran statistics are calculated in RAMPAGE.[12]

Beamline, Date of data collection	ID30A-3, ESRF, Grenoble; 18/02/2022
Autoprocessed dataset used	autoPROC
Wavelength [Å]	0.9762
Space group	*C2*
No. of mols/asymmetric unit	3
Cell parameters	
(a, b, c)[Å]	152.4, 52.2, 88.9
(α,β,γ)[°]	90.0, 105.7, 90.0
Resolution [Å]	85.6 – 2.65 (2.69 – 2.65)*
Completeness [%]	99.1 (99.7)
R_{meas} [%]	12.1 (71.3)
R_{pim} [%]	6.0 (34.6)
I/ σ(I)	10.5 (2.4)
$CC_{1/2}$ [%]	99.4 (75.9)
Observed reflections	78429 (4018)
Unique reflections	19765 (978)
Redundancy	4.0 (4.1)
R_{work} [%]	17.76
R_{free} [%]	23.33
RMSD	
Bond lengths [Å]	0.0082
Bond Angles [°]	1.6596
Ramachandran Statistics #(%)	
Favored	98.5
Allowed	0.8
Outlier	0.6

Table S2. Carbohydrates tested for screening *Mt*AA16A activity under different conditions

Substrate	Substrate concentration (mg/mL)	*Mt*AA16A concentration (μg/mg substrate)	Reducing agent	Temperature (°C)	H_2O_2 addition
RAC	2	10	Asc	50	-
	2	20	Asc	30	-
	2	20	Asc	30	50 μM
	2	20	Syr	30	-
	2	20	Syr	30	50 μM
	2	20	Gua	30	-
	2	20	Gua	30	50 μM
Avicel PH-101	2	20	Asc	30	-
	2	20	Asc	30	50 μM
Planetary ball milled Avicel PH-101	2	20	Asc	30	-
	2	20	Asc	30	50 μM
β-chitin from shrimp shell	2	10	Asc	50	-
	2	20	Asc	30	50 μM
β-chitin nano fiber	2	10	Asc	50	-
	2	20	Asc	30	50 μM
Regenerated amorphous β-chitin	5	30	Asc	30	-
	5	30	Asc	30	50 μM
Planetary ball milled β-chitin	5	30	Asc	30	-
	5	30	Asc	30	50 μM
β-chitosan	5	30	Asc	30	-
	5	30	Asc	30	50 μM
Regenerated amorphous β-chitosan	5	30	Asc	30	-
	5	30	Asc	30	50 μM
Planetary ball milled β-chitosan	5	30	Asc	30	-
	5	30	Asc	30	50 μM
Xylan from birch wood	2	10	Asc	50	-
	2	20	Asc	30	-
	2	20	Asc	30	50 μM
	2	20	Syr	30	-
	2	20	Syr	30	50 μM
	2	20	Gua	30	-
	2	20	Gua	30	50 μM
Xyloglucan from tamarind seed	2	20	Asc	30	-
	2	20	Asc	30	50 μM
	2	20	Syr	30	-
	2	20	Syr	30	50 μM
	2	20	Gua	30	-
	2	20	Gua	30	50 μM
Mannan from cassia	2	30	Asc	30	-
	2	30	Asc	30	50 μM
Galactan from potato	2	30	Asc	30	-
	2	30	Asc	30	50 μM
Laminarin from *Laminaria*	2	30	Asc	30	-

Substrate	Substrate concentration (mg/mL)	*Mt*AA16A concentration (μg/mg substrate)	Reducing agent	Temperature (°C)	H_2O_2 addition
digitata	2	30	Asc	30	50 μM
Glucomannan from konjac	2	30	Asc	30	-
	2	30	Asc	30	50 μM
Arabinan from sugar beet	2	30	Asc	30	-
	2	30	Asc	30	50 μM
Pectin (DM30) from lemon[a]	2	10	Asc	50	-
	2	20	Asc	30	-
	2	20	Asc	30	50 μM
Pectin (DM96) from lemon[b]	2	10	Asc	50	-
	2	20	Asc	30	-
	2	20	Asc	30	50 μM
Peptidoglycan from *Bacillus subtilis*	2	40	Asc	30	-
	2	40	Asc	30	50 μM
	2	40	Pyg	30	-
	2	40	Pyg	30	50 μM
Peptidoglycan from *Saccharomyces cerevisiae*	2	40	Asc	30	-
	2	40	Asc	30	50 μM
	2	40	Pyg	30	-
	2	40	Pyg	30	50 μM
Soy soluble polysaccharides	5	20	Asc	30	-
	5	20	Asc	30	50 μM
Fungal cell wall polysaccharides from *Pleurotus ostreatus*	5	20	Asc	30	-
	5	20	Asc	30	50 μM
RAC + Xylan	2 each	10	Asc	50	-
	2 each	20	Asc	30	-
	2 each	20	Asc	30	50 μM
	2 each	20	Syr	30	-
	2 each	20	Syr	30	50 μM
	2 each	20	Gua	30	-
	2 each	20	Gua	30	50 μM
RAC + Xyloglucan	2 each	10	Asc	50	-
	2 each	20	Asc	30	-
	2 each	20	Asc	30	50 μM
	2 each	20	Syr	30	-
	2 each	20	Syr	30	50 μM
	2 each	20	Gua	30	-
	2 each	20	Gua	30	50 μM
RAC + Pectin (DM30)	2 each	10	Asc	50	-
	2 each	20	Asc	30	-
	2 each	20	Asc	30	50 μM
RAC + Pectin (DM96)	2 each	10	Asc	50	-
	2 each	20	Asc	30	-

[a]DM30: Degree of methylation of 30; [b]DM96: Degree of methylation of 96

Table S3. Geometry at the *Mt*AA16A copper binding site. Angle definitions are as in Vu et al.[13]

Sample Name	Cu-Nam, Cu-ND1, Cu-NE2 (Å)	Cu-O_{Tyr} (Å)	θ_1, θ_2, θ_3 (°)	θ_T (°)	RMSD T (Å)	$\theta_{H\text{-}H}$ (°)	RMSD H1 (Å)	θ_{H1} (°)	RMSD HN(Å)	θ_{HN} (°)	Nam-Cu-O_{Tyr} (°)	ND1-Cu-O_{Tyr} (°)	NE2-Cu-O_{Tyr} (°)
MtAA16_chainA	2.18, 1.78, 1.98	2.70	98.99, 94.81, 162.62	10.6	0.06644	77.78	0.029	2.25	0.017	3.55	89.93	97.93	92.60
MtAA16_chainB	2.27, 1.86, 1.94	2.71	96.53, 99.33, 163.71	13.0	0.067	71.82	0.027	7.35	0.019	-2.97	85.56	104.73	88.91
MtAA16_chainC	2.25, 1.83, 1.91	2.67	96.21, 93.89, 165.62	10.2	0.075	78.45	0.030	5.04	0.017	3.35	88.91	101.85	88.53

Table S4. H_2O_2 producing activity of *Mt*AA16A, *Mt*LPMO9s and *Nc*LPMO9s in the presence of 50 μM Asc. H_2O_2 producing activity of *Mt*AA16A, *Mt*LPMO9s and *Nc*LPMO9s in the presence of 1 mM Asc is shown in Table 4. *See Experimental Section for assay conditions.

	H_2O_2 producing activity (mU)*
Cu(II) only	<0.1
Boiled-*Mt*AA16A	<0.1
*Mt*AA16A	1.51 ± 0.02
*Mt*LPMO9B	0.13 ± 0.01
*Mt*LPMO9E	<0.1
*Mt*LPMO9H	0.18 ± 0.01
*Mt*LPMO9I	0.69 ± 0.06
*Nc*LPMO9C	4.26 ± 0.12
*Nc*LPMO9F	0.68 ± 0.00
*Nc*LPMO9M	2.83 ± 0.14

References

1. Breslmayr, E.; Hanzek, M.; Hanrahan, A.; Leitner, C.; Kittl, R.; Santek, B.; Oostenbrink, C.; Ludwig, R., A fast and sensitive activity assay for lytic polysaccharide monooxygenase. *Biotechnol. Biofuels* **2018,** *11*, 79.
2. Sun, P.; Valenzuela, S. V.; Chunkrua, P.; Pastor, F. I. J.; Laurent, C. V. F. P.; Ludwig, R.; van Berkel, W. J. H.; Kabel, M. A., Oxidized product profiles of AA9 lytic polysaccharide monooxygenases depend on the type of cellulose. *ACS Sustain. Chem. Eng.* **2021,** *9*, 14124-14133.
3. Negi, S. S.; Schein, C. H.; Oezguen, N.; Power, T. D.; Braun, W., InterProSurf: a web server for predicting interacting sites on protein surfaces. *Bioinformatics* **2007,** *23*, 3397-3399.
4. van Zundert, G. C. P.; Rodrigues, J. P. G. L. M.; Trellet, M.; Schmitz, C.; Kastritis, P. L.; Karaca, E.; Melquiond, A. S. J.; van Dijk, M.; de Vries, S. J.; Bonvin, A. M. J. J., The HADDOCK2.2 web server: User-friendly integrative modeling of biomolecular vomplexes. *J. Mol. Biol.* **2016,** *428*, 720-725.
5. Teufel, F.; Almagro Armenteros, J. J.; Johansen, A. R.; Gíslason, M. H.; Pihl, S. I.; Tsirigos, K. D.; Winther, O.; Brunak, S.; von Heijne, G.; Nielsen, H., SignalP 6.0 predicts all five types of signal peptides using protein language models. *Nat. Biotechnol.* **2022,** *40*, 1023-1025.
6. Katoh, K.; Standley, D. M., MAFFT multiple sequence alignment software version 7: improvements in performance and usability. *Mol. Biol. Evol.* **2013,** *30*, 772-780.
7. Kumar, S.; Stecher, G.; Tamura, K., MEGA7: Molecular evolutionary genetics analysis version 7.0 for bigger datasets. *Mol. Biol. Evol.* **2016,** *33*, 1870-1874.
8. Holm, L., Using DALI for protein structure comparison. *Methods Mol. Biol.* **2020,** *2112*, 29-42.
9. Holm, L.; Rosenstrom, P., DALI server: conservation mapping in 3D. *Nucleic Acids Res.* **2010,** *38*, W545-W549.
10. Lassmann, T., Kalign 3: multiple sequence alignment of large datasets. *Bioinformatics* **2020,** *36*, 1928-1929.
11. Waterhouse, A. M.; Procter, J. B.; Martin, D. M. A.; Clamp, M.; Barton, G. J., Jalview Version 2: A multiple sequence alignment editor and analysis workbench. *Bioinformatics* **2009,** *25*, 1189-1191.
12. Lovell, S. C.; Davis, I. W.; Arendall III, W. B.; De Bakker, P. I.; Word, J. M.; Prisant, M. G.; Richardson, J. S.; Richardson, D. C., Structure validation by Cα geometry: ϕ, ψ and Cβ deviation. *Proteins: Struct. Funct. Genet.* **2003,** *50*, 437-450.
13. Vu, V. V.; Ngo, S. T., Copper active site in polysaccharide monooxygenases. *Coord. Chem. Rev.* **2018,** *368*, 134-157.
